# Estimating Gene Flow between Refuges and Crops: A Case Study of the Biological Control of *Eriosoma lanigerum* by *Aphelinus mali* in Apple Orchards

**DOI:** 10.1371/journal.pone.0026694

**Published:** 2011-11-02

**Authors:** Blas Lavandero, Christian C. Figueroa, Pierre Franck, Angela Mendez

**Affiliations:** 1 Laboratorio de Interacciones Insecto-Planta, Universidad de Talca, Talca, Chile; 2 Plantes et Systèmes de culture Horticoles, INRA, Avignon, France; 3 Facultad de Ciencias, Instituto de Ecología y Evolución, Universidad Austral de Chile, Valdivia, Chile; Netherlands Institute of Ecology, Netherlands

## Abstract

Parasitoid disturbance populations in agroecosystems can be maintained through the provision of habitat refuges with host resources. However, specialized herbivores that feed on different host plants have been shown to form host-specialized races. Parasitoids may subsequently specialize on these herbivore host races and therefore prefer parasitizing insects from the refuge, avoiding foraging on the crop. Evidence is therefore required that parasitoids are able to move between the refuge and the crop and that the refuge is a source of parasitoids, without being an important source of herbivore pests. A North-South transect trough the Chilean Central Valley was sampled, including apple orchards and surrounding *Pyracantha coccinea* (M. Roem) (Rosales: Rosacea) hedges that were host of *Eriosoma lanigerum* (Hemiptera: Aphididae), a globally important aphid pest of cultivated apples. At each orchard, aphid colonies were collected and taken back to the laboratory to sample the emerging hymenopteran parasitoid *Aphelinus mali* (Hymenoptera: Aphelinidae). Aphid and parasitoid individuals were genotyped using species-specific microsatellite loci and genetic variability was assessed. By studying genetic variation, natural geographic barriers of the aphid pest became evident and some evidence for incipient host-plant specialization was found. However, this had no effect on the population-genetic features of its most important parasitoid. In conclusion, the lack of genetic differentiation among the parasitoids suggests the existence of a single large and panmictic population, which could parasite aphids on apple orchards and on *P. coccinea* hedges. The latter could thus comprise a suitable and putative refuge for parasitoids, which could be used to increase the effectiveness of biological control. Moreover, the strong geographical differentiation of the aphid suggests local reinfestations occur mainly from other apple orchards with only low reinfestation from *P. cocinnea* hedges. Finally, we propose that the putative refuge could act as a source of parasitoids without being a major source of aphids.

## Introduction

Natural enemies of insect pests are constantly disturbed in agroecological systems, and classical management practices can severely reduce parasitoid populations. The use of habitat refuges, offering shelter and alternative hosts for these organisms, has been proposed for maintaining high density of parasitoids close to cultivated plants, acting as a constant source to control agricultural pests [1]. At larger scales, landscape heterogeneity has been proposed to have a positive effect on natural enemy populations and parasitism rates in general [2]. Nevertheless, one must have enough evidence that parasitoids do disperse between the refuges and the crop, and that they exert an effect on the herbivore populations.

Ecological specialization of herbivore insects could affect their relationship with the third trophic level. Specialist herbivores that feed on different host plants have been shown to form host-specialized races, evidenced through reduced migration and gene flow [3]. The effect on the next trophic level (the natural enemies) can follow the specialization of their herbivore host, resulting in the formation of specialized parasitoid races, in a process termed sequential radiation [4]. In fact, as herbivorous insects and their parasitoids interact with their environment on a fine spatial and temporal scale, sequential radiation may be quite common [5]. Thus, parasitoids coming from a refuge may not readily forage on the crop or they may be totally isolated if gene flow between the refuge and the crop is absent, in which case the refuge would not constitute a real source of parasitoids for improving biocontrol.

Genetic markers, particularly highly polymorphic ones such as microsatellites, have been widely used to study several aspects of insect ecology. These DNA markers provide the raw data to estimate genetic diversity and gene flow between insect populations or to reconstruct migration routes and colonization history. Using appropriate bioinformatic tools to analyze DNA marker data, gene flow and genetic diversity within insect species can be quantified, which is critical for explaining population structure and dynamics in time and space (for a review see [6]). For instance, microsatellites in combination with powerful analytical tools [7] have proven to be useful for describing movement of insect pests between continents (for the western corn rootworm see [8]; for the tobacco aphid see [9]), between different production areas (for the codling moth see Fuentes–Contreras et al. [10]; for the woolly apple aphid see [11]), and between native and introduced ranges of parasitoids [12]. To our knowledge, however, there are no studies using neutral genetic variation to estimate natural enemy migration (movement and reproduction) between a putative refuge and the crop.

Here, using neutral genetic variation, we show the existence of geographical natural barriers to aphids in a main apple production area. The level of host specialization of this aphid pest is shown to have no influence on the population differentiation of its most important parasitoid wasp, due to the high gene flow observed among plant species and locations. We argue that the proposed refuge could act as a source of parasitoids without being a major source of the aphid pest.

## Results

### The aphids

Aphids were found in apple orchards and at four *P. coccinea* hedge sites, irrespective of pest management practices (organic vs. conventional orchards) (Table 1). A total of 581 aphid colonies were sampled and 471 different multilocus genotypes characterized (for a list of multilocus genotypes see Table S1). Twenty six genotypes were found more than once. Frequency of these multicopy genotypes was low in most sites (less than 10%), with the exception of site *Cato* where 44.8% of the colonies belonged to the same genotype. The genotypic diversity was high and similar among all sites as evidenced by the indices of Shannon, Simpson and their evenness (Table 1). Mean standardized allelic richness per site varied from 2.7 to 4.1. Heterozygosity ranged between 0.68 and 0.95 and gene diversity between 0.53 and 0.71 (see Table 1). Significant and frequent departures from Hardy-Weinberg Equilibrium were found in most of the sampled sites due to heterozygote excess. No evidence for null alleles was found (data not shown).

**Table 1:** Site Number, Location, Host plant, Management conditions (O = Organic, C = Conventional), sample size, Number of Genotypes, Unique vs. Multicopy genotypes (U/M), Shannon diversity (H) and its evenness (VH), Simpson diversity (D) and its evenness (ED), Gene Diversity (1-Q), Inbreeding coefficient (*Fis*) and significance (p-value), Loci under disequilibrium and allelic richness (A) of *Eriosoma lanigerum* females per site.

Site N°	Location	Host plant	Manag.	n	Genotypes	U/M	H	VH	D	ED	(1-Q)	*Fis*	p-value	LD	A
1	Villa Alemana	*Malus*	O	30	28	26/2	3,309	0,993	0,995	0,519	0,867	−0,349	>0,01	2/22	3,6
2	Graneros	*Malus*	C	19	13	11/2	2,347	0,915	0,924	0,481	0,895	−0,642	>0,01	3/22	3
3	Cañadilla	*Malus*	O	13	13	13/0	2,565	1,000	1,000	−1,000	0,890	−0,492	>0,01	1/22	3,1
4	San Fernando	*Malus*	O	29	29	29/0	3,367	0,999	1,000	−1,000	0,823	−0,267	>0,01	4/22	3,4
5	Los Niches	*Malus*	O	51	50	49/1	3,905	0,998	0,999	0,000	0,790	−0,189	>0,01	7/22	3,7
6	Panguilemo	*Malus*	C	28	24	23/1	3,045	0,958	0,974	0,000	0,888	−0,460	>0,01	2/22	3,3
7	Maiten Huapi	*Malus*	O	58	55	53/2	3,980	0,993	0,998	0,453	0,867	−0,316	>0,01	5/22	3,7
8	Las Rastras	*Malus*	C	30	27	25/2	3,245	0,985	0,991	0,462	0,895	−0,351	>0,01	3/22	3,7
9	Colin	*Malus*	C	30	27	25/2	3,245	0,985	0,991	0,462	0,805	−0,256	>0,01	4/22	3,4
10	Las Lomas	*Malus*	C	27	27	27/0	3,296	1,000	1,000	−1,000	0,783	−0,185	>0,01	1/22	3,8
11	Pataguas	*Malus*	C	30	18	13/5	2,691	0,931	0,949	0,824	0,867	−0,554	>0,01	2/22	2,9
12	Miraflores	*Malus*	C	30	28	27/1	3,291	0,988	0,993	0,000	0,810	−0,219	>0,01	8/22	3,8
13	Ancoa	*Malus*	C	37	36	35/1	3,573	0,997	0,998	0,000	0,865	−0,329	>0,01	2/22	3,7
14	Huaquivilo	*Malus*	O	36	35	34/1	3,545	0,997	0,998	0,000	0,679	−0,070	NS	5/22	3,8
15	Miraríos	*Malus*	O	26	26	26/0	3,258	1,000	1,000	−1,000	0,769	−0,113	NS	10/22	4,1
16	Cato	*Malus*	C	29	12	9/3	1,913	0,770	0,786	0,498	0,828	−0,592	>0,01	13/22	2,8
17	Mulchén	*Malus*	O	28	26	25/1	3,214	0,987	0,992	0,000	0,745	−0,245	>0,01	3/22	3,4
			**SUBTOTAL**	**531**	**474**	**451/26**	**6,072**	**0,984**	**0,999**	**0,930**	**0,830**		**>0,001**	**19/22**	
A	Cañadilla	*Pyracantha*	n/a	12	7	5/2	1,748	0,898	0,864	0,560	0,917	−0,682	>0,001	0/22	2,7
B	Las Rastras	*Pyracantha*	n/a	5	5	5/0	1,609	1,000	1,000	−1,000	0,971	−0,432	>0,001	0/22	3,9
C	Colin	*Pyracantha*	n/a	19	19	19/0	2,944	1,000	1,000	−1,000	0,895	−0,397	>0,001	1/22	3,5
D	Manzano	*Pyracantha*	n/a	8	6	5/1	1,667	0,931	0,893	0,000	0,929	−0,526	>0,001	3/22	3,3
			**SUBTOTAL**	**44**	**37**	**34/3**	**3,508**	**0,972**	**0,987**	**0,671**	**0,928**		**>0,001**	**1/22**	
			**Whole sample**	**575**	**511**	**485/29**	**6,146**	**0,985**	**0,999**	**0,941**	**0,879**				

**Figure 1:**
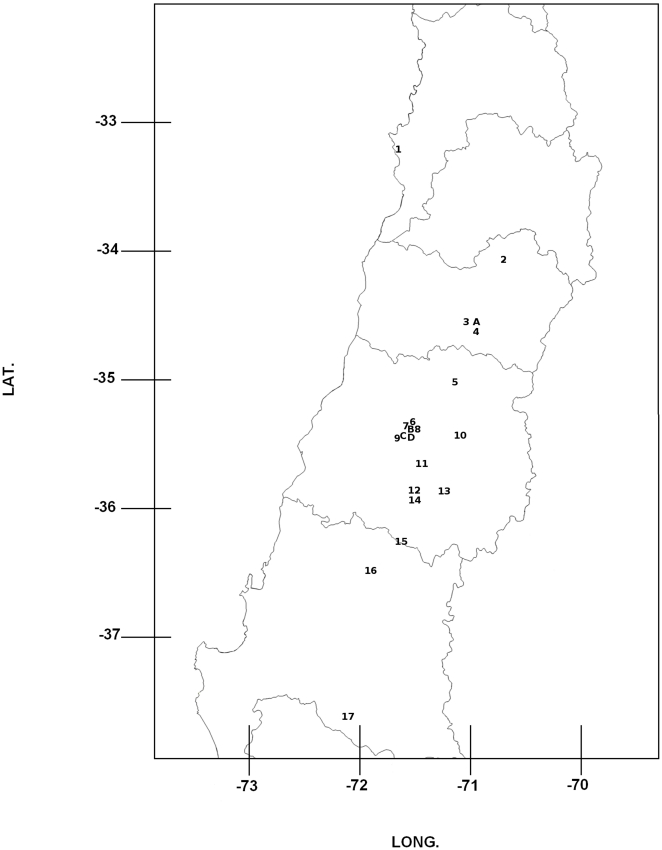
Collection sites of apple orchards (*Malus domestica*) (numbers) and *Pyracantha coccinea* sampling sites (letters). 1 Villa Alemana, 2 Graneros, 3 Cañadilla, 4 San Fernando, 5 Los Niches, 6 Panguilemo, 7 Maiten Huapi, 8 Las Rastras, 9 Colin, 10 Las Lomas, 11 Pataguas 12 Miraflores, 13 Ancoa, 14 Huaquivilo, 15 Miraríos, 16 Cato, 17 Mulchén, A Cañadilla, B Las Rastras, C Colin, D Manzano.

**Figure 2:**
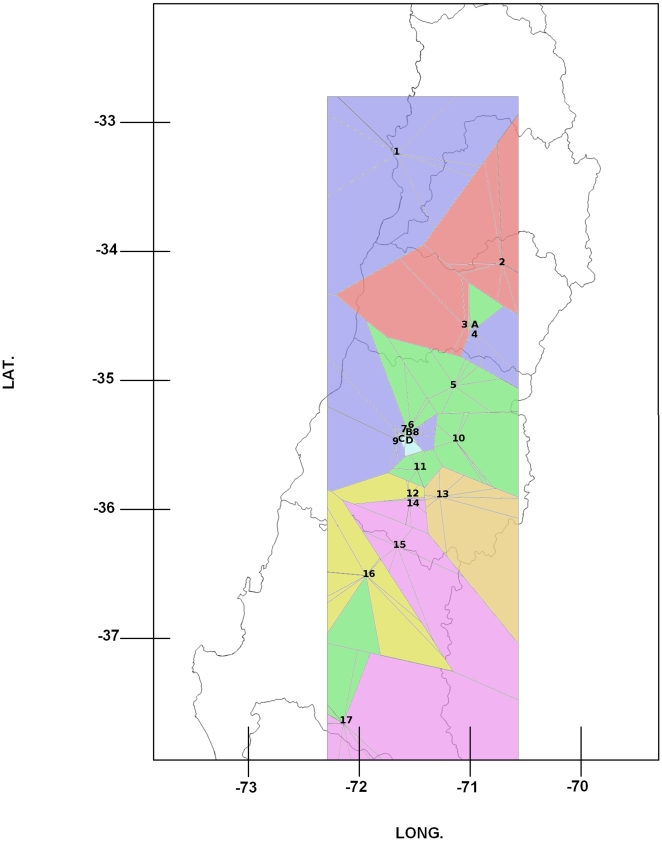
Membership of individuals of *Eriosoma lanigerum* based on 50,000 sweeps using TESS, assuming no admixis, between sites. Tessellation is ordered from North to South.

**Figure 3:**
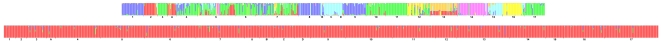
Average assignment probability of individuals of *Eriosoma lanigerum* (aphid host), independent of sampling origin. Assignment is based on 100 repetitions of 50,000 sweeps using TESS showing K = 7 genetic clusters and the correspondent structure for its parasitoid *Aphelinus mali*. Individuals (bars) are from North to South.

**Figure 4:**
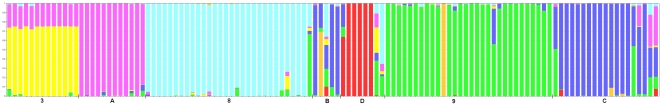
Average assignment probability of a subsample of individuals of *Eriosoma lanigerum* (aphid host), independent of sampling origin, based on 100 repetitions of 50,000 sweeps using TESS showing K = 7 genetic clusters, ordered showing neighbouring sites between both host plants (*Malus domestica* and *Pyracantha coccinea*). 3 = Cañadilla-*Malus*, A = Cañadilla-*Pyracantha*, 8 = Las Rastras-*Malus*, B = Las Rastras-*Pyracantha*, D = Los Manzanos–*Pyracantha*, 9 = Colin-*Malus*, C = Colin-*Pyracantha*.

**Figure 5:**
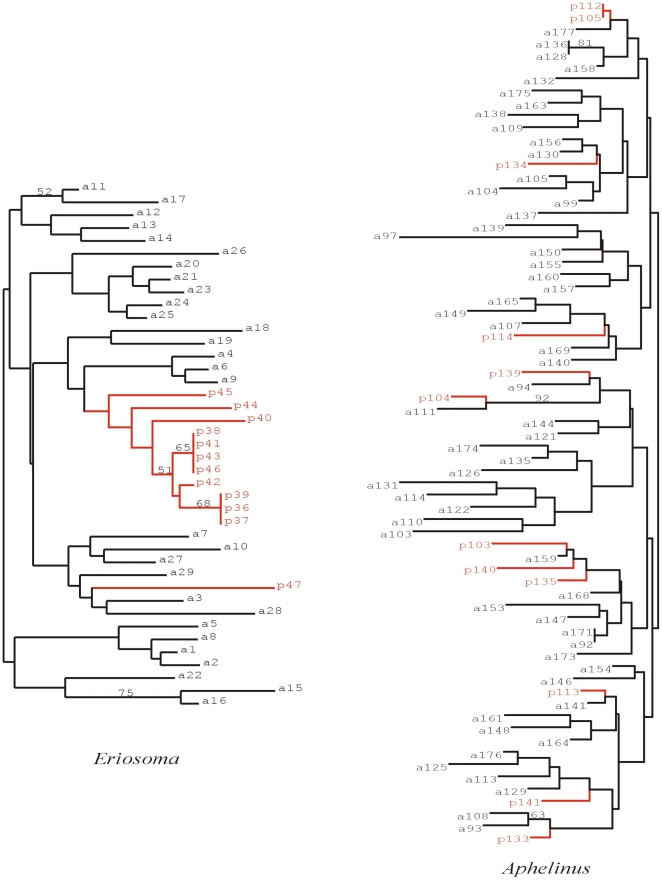
A neighbor-joining tree constructed using the shared allele distance between individuals of a single site (*Cañadilla*, site 3 **Figure 1**) among individuals collected on different host plants *Pyracantha* (in red) and *Malus* (black). Bootstrap values were computed over 2000 replications resampling the microsatellite loci. On the left side the tree for the aphid *Eriosoma lanigerum* and on the right the tree for the emerged parasitoids (*Aphelinus mali*).

The genetic differentiation of populations (*Phi-pt*) between sites ranged from 2 to 23%. Analyses of Molecular Variance (AMOVA) of the aphid populations revealed different genetic structures that can be explained both by differences among the sites (22%) and differences between the host plants (2%) (p = 0.01). Pairwise comparisons between pairs of neighbouring *Pyracantha* hedges and their corresponding apple orchards showed a significantly high differentiation, ranging between 12.3% for *Colin* (site 9 and C, Figure 1) and 39% for *Cañadilla* (site 3 and A, Figure 1). Further analyses using TESS suggested that the aphid colonies were grouped into seven geographically related clusters, where sites close to each other shared more ancestry than those further apart (represented in Figure 2 and 3 (top) by different colours). The Bayesian clustering method showed different genetic clusters between neighbouring collection sites including samples from different host plants. This was confirmed after analyzing a smaller comparable scale (*P. coccinea* sites A, B, C and D; Apple sites 3, 8 and 9, in Figure 1), revealing a high differentiation between host-plants (5%; *p* = 0.01), although the greater differences among populations were independent of the host (21%; *p* = 0.01). Further analyses using TESS confirmed the AMOVA results by showing almost no admixis between host plants or sites (Figure 4). Analyses using shared allelic distance between individuals at the site *Cañadilla* suggest that aphids from the same host plant are more closely related (Figure 5).

### The parasitoids

A total of 1018 parasitoid specimens were obtained (one to three parasitoids emerged from each aphid colony sampled) and 902 individuals were successfully genotyped and considered for analyses. Mean standardized allelic richness per site varied from 3.1 to 4.0. Allelic richness of the parasitoid was independent of the geographical distance between sites (Partial Mantel test; r = −0.1, *p* = 0.46). The proportion of heterozygotes ranged between 0.26 and 0.50, while gene diversity ranged between 0.39 and 0.54 (see Table 2). Slight heterozygote deficiencies were detected in most sites, probably due to null alleles (frequency of null alleles was under 19% for all loci). AMOVA evidenced significant but very low variation between sites (1%) and within host plants (1%), suggesting great gene flow between sites and host plants at the landscape level (see further details of pairwise *Fst* in Table 3). Further analyses using the Bayesian structuring algorithm implemented in TESS and considering all individuals independent of their collection sites, suggested no host or geographically-associated differentiation for the parasitoids (see Figure 3).

**Table 2:** Management conditions, Host plant, Parasitism rates, allelic richness, Observed Heterozygocity (Ho) and Gene Diversity of *Aphelinus mali* females per site.

				Parasitism rates		Samples	Allelic Richness		Ho		Gene diversity	
Site N°	Location	Management	Host plant	mean	SD	N	Mean	SD	Mean	SD	Mean	SD
1	Villa Alemana	O	*Malus*	70%	48%	16	4,1	0,9	0,28	0,07	0,52	0,11
2	Graneros	C	*Malus*	95%	31%	15	3,4	1,2	0,31	0,13	0,42	0,21
3	Cañadilla	O	*Malus*	79%	47%	27	3,4	0,8	0,33	0,14	0,52	0,21
4	San Fernando	O	*Malus*	73%	26%	61	3,8	1,0	0,35	0,15	0,49	0,16
5	Los Niches	O	*Malus*	82%	42%	60	3,7	1,1	0,38	0,15	0,53	0,23
6	Panguilemo	C	*Malus*	96%	9%	70	3,7	1,1	0,39	0,14	0,49	0,19
7	Maiten Huapi	O	*Malus*	95%	9%	66	3,7	1,2	0,40	0,16	0,53	0,19
8	Las Rastras	C	*Malus*	88%	18%	21	3,1	1,1	0,38	0,19	0,39	0,15
9	Colin	C	*Malus*	94%	14%	48	4,0	1,0	0,38	0,18	0,49	0,19
10	Las Lomas	C	*Malus*	91%	31%	65	4,0	1,0	0,37	0,15	0,51	0,20
11	Fundo Pataguas	C	*Malus*	96%	13%	57	3,7	1,1	0,36	0,13	0,50	0,16
12	Miraflores	C	*Malus*	67%	50%	29	3,9	1,4	0,34	0,11	0,52	0,17
13	Ancoa	C	*Malus*	100%	/	72	3,6	1,2	0,31	0,11	0,46	0,15
14	Huaquivilo	O	*Malus*	86%	40%	49	3,7	1,2	0,37	0,12	0,48	0,18
15	Miraríos	O	*Malus*	84%	32%	24	4,1	0,8	0,50	0,18	0,51	0,17
16	Cato	C	*Malus*	94%	15%	56	4,0	1,3	0,42	0,20	0,51	0,22
17	Mulchén	O	*Malus*	93%	23%	73	3,9	1,3	0,43	0,17	0,54	0,16
A	Cañadilla	-	*Pyracantha*	100%	/	12	4,0	1,0	0,26	0,09	0,48	0,12
B	Las Rastras	-	*Pyracantha*	100%	/	18	3,5	1,3	0,33	0,12	0,54	0,18
C	Colin	-	*Pyracantha*	100%	/	29	3,7	1,2	0,39	0,16	0,45	0,22
D	Manzanos	-	*Pyracantha*	100%	/	22	3,8	0,9	0,33	0,15	0,46	0,20
			*Pyracantha*									
	Mean			90%			3,7	1,1	0,36	0,14	0,49	0,18

**Table 3:** Pairwise Population *Fst* Values, *Aphelinus mali*.

	Pop1	Pop2	Pop3	Pop4	Pop5	Pop6	Pop7	Pop8	Pop9	Pop10	Pop11	Pop12	Pop13	Pop14	Pop15	Pop16	Pop17	Pop18	Pop19	Pop20	Pop21
**Pop1**		0.460	0.110	0.010	0.010	0.430	0.270	0.450	0.300	0.140	0.050	0.290	0.030	0.430	0.290	0.290	0.170	0.070	0.510	0.260	0.380
**Pop2**	0.000		0.270	0.010	0.160	0.210	0.030	0.010	0.030	0.050	0.030	0.030	0.020	0.300	0.040	0.020	0.390	0.010	0.280	0.150	0.390
**Pop3**	0.005	0.002		0.010	0.160	0.010	0.020	0.010	0.140	0.040	0.050	0.060	0.010	0.060	0.120	0.110	0.460	0.010	0.060	0.010	0.370
**Pop4**	0.034	0.034	0.031		0.020	0.010	0.020	0.010	0.010	0.040	0.040	0.010	0.020	0.020	0.010	0.030	0.010	0.010	0.020	0.010	0.010
**Pop5**	0.022	0.008	0.006	0.042		0.010	0.010	0.010	0.030	0.010	0.010	0.030	0.010	0.010	0.010	0.010	0.430	0.010	0.170	0.010	0.160
**Pop6**	0.000	0.002	0.014	0.043	0.038		0.310	0.420	0.030	0.040	0.200	0.180	0.010	0.390	0.180	0.020	0.040	0.010	0.200	0.510	0.060
**Pop7**	0.004	0.014	0.009	0.029	0.047	0.000		0.170	0.010	0.140	0.470	0.470	0.520	0.260	0.450	0.040	0.010	0.100	0.020	0.020	0.040
**Pop8**	0.000	0.012	0.017	0.035	0.043	0.000	0.004		0.010	0.020	0.060	0.080	0.010	0.190	0.410	0.020	0.010	0.030	0.160	0.090	0.040
**Pop9**	0.002	0.006	0.003	0.054	0.022	0.012	0.015	0.015		0.010	0.010	0.030	0.010	0.020	0.310	0.040	0.490	0.010	0.020	0.010	0.450
**Pop10**	0.006	0.007	0.007	0.022	0.024	0.009	0.007	0.014	0.024		0.120	0.040	0.090	0.310	0.010	0.210	0.010	0.010	0.110	0.050	0.070
**Pop11**	0.010	0.015	0.015	0.027	0.060	0.004	0.000	0.013	0.019	0.008		0.170	0.470	0.050	0.500	0.070	0.020	0.010	0.020	0.050	0.060
**Pop12**	0.002	0.010	0.009	0.044	0.027	0.002	0.000	0.005	0.009	0.011	0.007		0.430	0.160	0.070	0.010	0.050	0.030	0.080	0.010	0.100
**Pop13**	0.010	0.016	0.015	0.034	0.036	0.010	0.000	0.018	0.021	0.005	0.000	0.000		0.040	0.030	0.020	0.010	0.020	0.030	0.020	0.020
**Pop14**	0.000	0.001	0.007	0.038	0.026	0.000	0.003	0.003	0.010	0.001	0.012	0.004	0.008		0.200	0.380	0.040	0.440	0.240	0.230	0.160
**Pop15**	0.000	0.012	0.006	0.025	0.045	0.005	0.000	0.000	0.003	0.012	0.000	0.009	0.012	0.004		0.320	0.020	0.020	0.030	0.020	0.240
**Pop16**	0.001	0.011	0.005	0.034	0.026	0.011	0.009	0.012	0.009	0.003	0.012	0.012	0.010	0.001	0.002		0.060	0.010	0.050	0.030	0.080
**Pop17**	0.008	0.000	0.000	0.065	0.001	0.019	0.031	0.029	0.000	0.035	0.042	0.021	0.032	0.019	0.025	0.017		0.010	0.260	0.010	0.400
**Pop18**	0.012	0.025	0.029	0.075	0.062	0.015	0.010	0.018	0.028	0.025	0.031	0.012	0.018	0.000	0.025	0.020	0.047		0.080	0.050	0.030
**Pop19**	0.000	0.002	0.010	0.050	0.012	0.007	0.026	0.008	0.015	0.015	0.036	0.009	0.022	0.004	0.026	0.012	0.006	0.020		0.150	0.320
**Pop20**	0.005	0.007	0.033	0.050	0.062	0.000	0.018	0.009	0.032	0.019	0.022	0.023	0.020	0.003	0.026	0.021	0.051	0.017	0.010		0.060
**Pop21**	0.000	0.000	0.002	0.058	0.010	0.015	0.034	0.026	0.000	0.027	0.023	0.019	0.025	0.010	0.009	0.014	0.000	0.035	0.002	0.024	

*Fst* Values below diagonal.

Probability values based on 9999 permutations are shown above diagonal.

Kinship analysis also detected numerous full-sib pairs between parasitoids collected from different aphid colonies sampled from either the same or different trees. Furthermore, parasitoid females emerging from the same aphid colony were usually not full-sibs (Table 4). Parasitism levels ranged from 67.3% to 100%, with no significant differences between organic or conventional orchards (*p* = 0.897). In contrast, parasitism levels were significantly higher on aphids collected from *P. coccinea* than those collected from apples (see Table 2).

**Table 4:** Percentage of parasitoid full sibs from the same aphid colony, from aphid colonies collected on the same host plant (for *Pyracantha* and *Malus*) and on different host plants at the Colin and Cañadilla sites.

	% full sibs same Colony	% full sibs *Pyracantha*	% full sibs *Malus*	% full sibs other Host
**Colin**	7.25	18.84	33.33	47.83
**Cañadilla**	18.32	2.29	68.70	29.01

### Aphid-parasitoid complex

Mean standardized allelic richness for the parasitoids per site were inversely correlated with the parasitism rates per orchard (Spearman r = −0.5, p = 0.038). Parasitism rates were independent of geographical distance when controlling for allelic richness (r = −0.11, p = 0.14). When estimating parasitism rates for the *Malus* sites per genetic cluster according to TESS (Mean ± SE: *Blue* 81.5±4.2; *Dark Yellow* 100±0; *Green* 91.8±3.03; *Pink* 87.7±4.02; *Red* 97.4±1.67 and *Yellow* 81.6±8.59), clusters *Blue* and *Yellow* (Figure 2) had significantly lower parasitism rates (Z values and correspondent p-values for paired comparisons with the *Blue* cluster for the *Dark Yellow z* = 6.266 *Green* z = 5.239 *Pink* z = 2.909; *Red* z = 6.303 and *Yellow* z = 0.001; *p* = 3.70e-10; *p* = 1.61e-07; *p* = 0.00363; *p* = 2.92e-10 and *p* = 0.99951).

Analyses using shared allelic distance between individuals at the site level for the populations from *Cañadilla* suggested that aphids from the same host plant were more closely related; however, the comparable tree for the parasitoids (constructed with individuals emerged from those same aphids), showed no significant grouping of parasitoids per tree or host plant (Figure 5).

## Discussion

The very low genetic differentiation among *A. mali* populations suggests that individuals do disperse between sites and host plants, although there is still no clear evidence that this can exert a difference in the herbivore abundances on the crop. The partitioning of molecular variance of the parasitoids revealed very low levels of variation between sites (i.e. orchards), especially considering that parasitoids reproduce sexually. Since no host or geographically-associated structuring was evident for the parasitoid, the natural barriers affecting aphids [11] seem not to be affecting the parasitoids. Moreover, the kinship analysis of parasitoids suggests that oviposition does not occur in a patchy or aggregated fashion. Thus, female parasitoids would lay eggs far away from each other, reducing the endogamy between points by increasing gene flow, at least at the orchard level, thus supporting the idea of a higher dispersal and gene flow between sites. Bayesian grouping algorithms revealed no geographic or host-driven structuring for the parasitoid, although the aphid host showed seven geographically related groups, where sites close to each other shared more ancestry than those further apart.

As reported before, aphids show low levels of gene flow at the landscape scale, with significant barriers between geographical areas [11]. The high levels of Heterozygosity, and few linked loci, suggest the occurrence of sexual reproduction in *E. lanigerum* in Chile, although this aphid species has not been found on its primary host where sexual reproduction is reported to occur (*Ulmus americana*) [13]. As suggested by Sandanayaka and Bus [14], sexual reproduction could indeed occur on apple, but further studies are necessary to determine the environmental conditions needed to trigger sexual reproduction, and to screen for the presence of sexual morphs in Chile. Interestingly, environmental conditions such as short days and below-zero temperatures (the factors that trigger sexuality in many aphid species [13], [15]), could affect parasitism rates through an increased genetic diversity in the aphid host. In any case, this seems not be enough to affect the parasitoids genetic structure.

The genetic diversity of the woolly apple aphid is clearly geographically structured; however, some of the genetic variation can be also be explained by the different host plants used by the aphids. Analyses comprising only those sites where neighbouring *Pyracantha* hedges are found, suggest a higher differentiation between host plants. Interestingly, the genetic clusters at each *Malus* site were different compared to their corresponding *Pyracantha* hedge. Evidence obtained from TESS, AMOVA and the neighbour-joining tree analyses, clearly separate individuals coming from different host plants. When the survival and preference of females were compared in reciprocal-transference experiments, *E. lanigerum* from *M. domestica* showed a stronger preference for its own natal branch as compared with other *M. domestica* or *P. coccinea* trees (Lavandero, unpublished data). In contrast, aphids born on *P. coccinea* had no significant preference for its natal host, showing a lower rejection for the *M. domestica* host. This could be the case for *E. lanigerum* aphids coming from *Malus*, which are not able to disperse into neighbouring *P. coccinea* hedges, although some individuals from *P. coccinea* may successfully colonize apple trees. This suggests that although *P. coccinea* could potentially become a source of some recolonizing aphids, it should not act as a significant source, as there seems to be a restricted and biased migration between both host plants. Hence, our results are indicative of no sequential radiation in this aphid-parasitoid system; however, aphids still exhibit geographical and some host-driven genetic structure.

Parasitism rates varied greatly among the studied sites; however, the management of the orchards (organic or conventional) did not explain these differences as expected. The literature suggests that the main explanation for parasitism decrease and aphid population outbreaks are due to the susceptibility of the parasitoids to pesticides (organophosphates and pyrethroids), sulphur and kaoline [16]–[18]. In both management systems, however, management practices alone cannot account for the differences found (67.3% to 100% rates of parasitism). Indeed, parasitism rates were not related with geographical distance between sites, even considering allelic richness, which could be used as an estimator of effective population sizes [19]. In our study, the allelic richness of the parasitoids was negatively correlated with the parasitism rates per site, which suggest inverse density dependence, meaning that parasitoids are effectively controlling the aphid populations up to a threshold where the rate of increase of aphid populations is greater than the parasitoid ability to exert control. The thermal biology of these organisms could explain this pattern, as the parasitoid has a greater thermal developmental threshold than its aphid host, translating into a lower growth rate (GR) compared to its host (GR = 0.1 parasitoid, 0.14–0.27 for the aphid at 20°C) [20]. On the other hand, aphid populations showed different genetic structures, some genetic clusters showing more susceptibility to *A. mali* parasitism than others, with no significant effect of management practices (i.e. genetic cluster grouped aphids coming from both conventional and organic orchards). Other factors such as land use and nectar availability for parasitoids, among others, need to be further analyzed, as well as the possible interaction between aphid and defense endosymbiont bacteria as found for other aphid species [21].

In conclusion, the lack of genetic differentiation of the parasitoids suggest the existence of a single large and panmictic population, which could parasitise aphids on apple orchards and on *P. coccinea* hedges, the latter being a suitable and putative refuge for parasitoids to increase their effectiveness in biological control. Moreover, the strong geographical differentiation of the aphid suggests that local reinfestations occur mainly from other apple orchards, with little reinfestation occurring from *P. coccinea* hedges. Further mark-recapture studies should be conducted to quantify dispersal, frequency and intensity of aphid infestations in apple orchards coming from both host plants. Quantification of the actual effect of this putative refuge on the population dynamics of the pest across several seasons will be critical if any effort for improving biocontrol is attempted using *P. coccinea*. Overall, we have shown that neutral genetic variation is a useful tool for addressing population dynamics between host plant species of pests and their parasitoids, determining potential refuges for natural enemies.

## Materials and Methods

### Study system

Aphids are important pests and disease vectors for a variety of crops, and parasitoids are often introduced for aphid biological control. The woolly apple aphid (*Eriosoma lanigerum* (Haussman)) (Hemiptera: Aphididae) native to North America, is a globally-important pest of apple orchards (*Malus domestica* Borkh). This aphid forms colonies on roots, trunks, branches and shoots, with greatest damage occurring at the shoot level [22]. Other associated damage is cosmetic, as fruits become covered with honeydew leading to subsequent fungus colonization, which reduces their commercial value. Although *M. domestica* is its most common host, this aphid also attacks other Rosacea species, notably *Pyracantha coccinea* (M. Roem) (Rosales: Rosacea), which is a very common plant distributed along farm hedges.

The wooly apple aphid (*E. lanigerum*) was first introduced into Chile during the 19^th^ century, most probably as root colonies from plant material. As the damage to apple orchards in Chile reached dramatic levels, in 1920 the chalcidoid parasitoid *Aphelinus mali* (Hymenoptera: Aphelinidae) was introduced. Although this parasitoid is the main species controlling *E. lanigerum* in Chile, it has been determined that under the current management conditions (conventional agriculture), aphid population outbreaks still occur [23]. There are several reasons for aphid population outbreaks, the most important probably being organophosphates, pyrethroids, sulfur and even kaolin treatments that affect its main parasitoid, *A. mali* [16]–[18]. In order to improve the effectiveness of the parasitoid, the use of host-plant refuges such as *Pyracantha coccinea* is proposed to attract and maintain parasitoid populations. Indeed, *E. lanigerum* is frequently observed at high densities on *P. coccinea*, with high parasitism rates by *A. mali*. This proposed refuge could be a source of parasitoids when the pest is not present in the orchard or as protection after pesticide use. However, evidence is required that the parasitoids are able to move between the refuge (*P. coccinea*) and the crop (apple), thereby determining its suitability as a source or sink for both aphids and parasitoids.

A 700 Km. North-South transect was sampled, including 17 apple orchards and surrounding *Pyracantha coccinea* hedges at four of the 17 chosen sites (33.19 S 71.733 W to 37.721 S 72.244 W). Orchards were all over 30 ha in size, planted with the Granny Smith apple cultivar. Permissions for entering and taking samples at conventional orchards were issued as part of an ongoing center within Universidad de Talca, Centro de Pomaceas (Stone fruit center), which gives the university the faculty of sampling in their farms (more details at http://pomaceas.utalca.cl/html/index.html). All orchards sampled are members of this center. Permission for entering and using materials of organic orchard were issued as part of an ongoing agreement between Comercial Greenvic Ltda and Universidad de Talca, through their branch Huertos Organicos de Chile S∶A: (more details at http://www.huertosorganicosdechile.cl/). All organic orchards sampled are members of this industry-university research agreement. At each orchard, up to 40 colonies of *E. lanigerum* were collected on different apple trees, while all available colonies on the *P. coccinea* hedges were sampled. Each aphid colony was georeferenced and taken back to the laboratory to determine parasitism rates under controlled conditions (20±1°C, 65±10% RH y 16∶8 hrs. day/night cycle). Parasitism rates per orchard were assessed for 10 trees (one colony per tree) per orchard. Colonies taken from the field were individually caged and reared under controlled conditions for two weeks. The number of aphids per colony and emerged parasitoids were registered from each cage. A single wingless adult aphid female per colony was preserved in 95% alcohol for subsequent DNA extraction. Parasitism rates were assessed by rearing aphids on 9 cm long shoots placed on a damp tissue paper inside plastic boxes with top ventilation. At emergence, parasitoids were identified to the species level, and up to three *A. mali* females per colony were preserved in 95% alcohol for DNA extraction. Genomic DNA was obtained following the ‘salting out’ protocol from [24]. Aphid and parasitoid individuals were genotyped using seven (aphids) and six (parasitoids) microsatellite (SSR) markers described in [25] and [26], respectively. The reverse primer for each pair of primers was fluorescently labeled, and PCR products analyzed on a MegaBASE 1000 automatic DNA Sequencer.

The microsatellite data were checked for null alleles and technical artifacts like stuttering bands and large allele dropout using the MICRO CHEKER v.2.2.3 software [27]. Deviations from the Hardy-Weinberg equilibrium (HWE) and linkage disequilibrium (LD) were tested using GENEPOP v.3.2a software [28]. To analyze genotypic data and test for clonality in the aphid populations, the number of genotypes, the rate of unique vs./multicopy genotypes, Shannon diversity and its evenness, Simpson diversity and its evenness, gene diversity, inbreeding coefficient (Fis) and significance (p-value), loci under disequilibrium and allelic richness per site were estimated using the GenClone 2.0 software [29]. Observed heterozygocity, gene diversity and allelic richness of *A. mali* per site were estimated using HP-RARE 1.0 [30]. Population structure of both species (parasitoids and aphids) was examined first using a hierarchical analysis of molecular variance (AMOVA) assuming asexuality for the aphids (*Phi-pt*; significant deviations from HWE) and sexuality for the parasitoids (*Fst*) as implemented in Genalex v 6.41 [31], with two levels (host plants and total effect). In addition, the population-genetic structure was assessed for aphids and parasitoids using the aggregation Bayesian algorithm implemented in TESS 2.3 [32]. The admixture model was compared with a non-admixture model as suggested by [33], because admixture models are robust to an absence of admixture in the sample, but non-admixture models are robust when admixture is present between some individuals. The TESS algorithm was run with 10,000 sweeps, discarding the first 5,000 with 20 independent iterations for each model for maximum clusters (Kmax) varying from 2 to 12 for both aphids and parasitoids. The highest likelihood runs were selected based on the Deviance Information Criterion (DIC) and graphed against Kmax (as suggested by [32]), allowing selection of the number of hypothetical clusters (K). Then the program was run 100 times for the selected Kmax with 50,000 sweeps discarding the first 10,000. The 10 highest likelihood runs were then averaged. Population genetic structure was assessed again on a subsample consisting of sampling sites with neighboring *P. coccinea* (sites 3, 8 and 9 for *Malus* and A, B, C and D for *P. coccinea* in Figure 1) using the aggregation Bayesian algorithm implemented in TESS, as described before. At the *Cañadilla* site (site 3 on Figure 1) a neighbor-joining tree [34] was constructed using the shared allele distance [35] between individuals, in order to visualize the genetic similarity among individuals collected on different host plants (*P. coccinea* and apple), as site 3 was the only site where aphids were found on hedges of *P. coccinea* inside an apple orchard. Bootstrap values were computed over 2000 resamplings of the microsatellite loci. In order to assess the ability of a parasitoid female to lay eggs grouped or dispersed among the aphid colonies, parasitoids that emerged from the same aphid colony were tested for being daughters from a single or many females. This was done using a kinship analysis on parasitoids that emerged from aphids sampled at sites where neighboring *P. coccinea* hedges are found (sites *Cañadilla* and *Colin*, 3 and 9 in Figure 1, respectively). Analyses were carried out using the full likelihood method [36], [37], as implemented in the software COLONY v 2.0, with data from six SSR loci.

In order to test the hypothesis that parasitoids respond to aphid population structure independently from geographical or sampling effects, a series of partial and simple Mantel tests were carried out. The significance of these correlations were assessed using *zt* version 1.0 [38], with 10.000 permutations [39]. The tested variables were parasitoid allelic richness as an estimate of population sizes, geographical distance between sites, sample size between sites, and parasitism rates per site. Spearman correlation was also carried out between parasitism rates per site and allelic richness of the parasitoids, using R version 2.10.1. Once the number of genetic clusters was estimated for the aphids, parasitism rates per cluster were estimated to asses the influence of the aphid's genetic background on the efficiency of the parasitoid. A generalized linear model (GLM) assuming a Poisson distribution was carried out [40] with the *glm* function in the base package of R version 2.10.1 written by Simon Davies. Mean values per cluster were then compared to the lowest mean value in a series of paired comparisons, and significances were estimated.

## Supporting Information

Table S1 **List of multilocus genotypes of sampled** ***Eriosoma lanigerum*** **.**
(DOCX)
