# Supplementary material for: Estimating Gene Flow between Refuges and Crops: A Case Study of the Biological Control of Eriosoma lanigerum by Aphelinus mali in Apple Orchards
Source: PLoS One. 2011 Nov 2;6(11):e26694. doi: 10.1371/journal.pone.0026694 (PMC3206839; doi:10.1371/journal.pone.0026694)
Supplement: Table S1 — List of multilocus genotypes of sampled Eriosoma lanigerum . (DOCX) [file pone.0026694.s001.docx]

**Table S1: List of multilocus genotypes of sampled *Eriosoma lanigerum*.**

|  |  |  | |  |  |  | |  |  |  |  | |  |  |  | |  |
| --- | --- | --- | --- | --- | --- | --- | --- | --- | --- | --- | --- | --- | --- | --- | --- | --- | --- |
| Sample | E3 |  | E20 |  | E29 |  | E33 |  | E72 |  | E75 |  | E78 |  |  | No. Matches | |
| colin1 | 150 | 165 | 176 | 179 | 186 | 189 | 174 | 180 | 160 | 160 | 142 | 150 | 165 | 169 |  | 1 | |
| colin4 | 153 | 153 | 167 | 170 | 186 | 189 | 174 | 180 | 160 | 166 | 142 | 150 | 165 | 169 |  | 1 | |
| colin5 | 153 | 153 | 176 | 179 | 189 | 189 | 177 | 180 | 160 | 162 | 142 | 150 | 169 | 179 |  | 1 | |
| colin6 | 153 | 153 | 176 | 179 | 186 | 186 | 174 | 180 | 158 | 162 | 150 | 164 | 169 | 179 |  | 1 | |
| colin7 | 150 | 165 | 176 | 179 | 189 | 189 | 174 | 180 | 160 | 166 | 144 | 150 | 165 | 169 |  | 1 | |
| colin8 | 153 | 165 | 176 | 179 | 186 | 189 | 174 | 180 | 162 | 166 | 144 | 164 | 165 | 179 |  | 1 | |
| colin9 | 150 | 165 | 176 | 179 | 189 | 189 | 174 | 180 | 160 | 166 | 142 | 142 | 165 | 169 |  | 1 | |
| colin12 | 150 | 165 | 176 | 179 | 189 | 189 | 174 | 180 | 160 | 166 | 142 | 150 | 165 | 169 |  | 3 | |
| colin13 | 153 | 153 | 167 | 179 | 186 | 186 | 174 | 180 | 166 | 170 | 142 | 150 | 171 | 173 |  | 1 | |
| colin14 | 150 | 153 | 167 | 179 | 189 | 189 | 174 | 180 | 158 | 162 | 142 | 150 | 173 | 173 |  | 1 | |
| colin15 | 153 | 165 | 167 | 179 | 186 | 189 | 174 | 180 | 162 | 166 | 144 | 150 | 165 | 173 |  | 1 | |
| colin16 | 150 | 165 | 167 | 170 | 189 | 189 | 174 | 180 | 160 | 166 | 142 | 142 | 165 | 169 |  | 1 | |
| colin17 | 153 | 153 | 176 | 179 | 186 | 189 | 174 | 180 | 158 | 162 | 142 | 150 | 165 | 169 |  | 2 | |
| colin18 | 153 | 153 | 176 | 179 | 186 | 189 | 174 | 180 | 158 | 166 | 144 | 150 | 165 | 169 |  | 1 | |
| colin19 | 150 | 165 | 167 | 179 | 189 | 189 | 174 | 180 | 160 | 166 | 144 | 150 | 165 | 169 |  | 1 | |
| colin20 | 150 | 165 | 167 | 179 | 189 | 189 | 177 | 177 | 166 | 170 | 142 | 150 | 165 | 169 |  | 1 | |
| colin21 | 153 | 165 | 170 | 176 | 186 | 189 | 180 | 180 | 160 | 166 | 150 | 164 | 165 | 169 |  | 1 | |
| colin22 | 150 | 165 | 167 | 179 | 186 | 189 | 174 | 180 | 160 | 166 | 142 | 164 | 165 | 169 |  | 1 | |
| colin23 | 150 | 153 | 176 | 179 | 186 | 186 | 174 | 180 | 158 | 162 | 144 | 164 | 165 | 173 |  | 1 | |
| colin24 | 150 | 165 | 176 | 179 | 186 | 186 | 174 | 180 | 162 | 166 | 144 | 150 | 165 | 173 |  | 1 | |
| colin25 | 165 | 165 | 167 | 179 | 186 | 186 | 174 | 180 | 160 | 166 | 142 | 142 | 165 | 173 |  | 1 | |
| colin26 | 153 | 165 | 176 | 179 | 186 | 186 | 174 | 180 | 162 | 166 | 150 | 150 | 162 | 179 |  | 1 | |
| colin27 | 150 | 153 | 167 | 179 | 186 | 189 | 180 | 180 | 160 | 166 | 142 | 142 | 165 | 169 |  | 1 | |
| colin28 | 150 | 165 | 167 | 179 | 186 | 189 | 180 | 180 | 160 | 166 | 142 | 150 | 165 | 169 |  | 1 | |
| colin29 | 150 | 165 | 167 | 179 | 186 | 186 | 177 | 180 | 162 | 162 | 142 | 150 | 165 | 169 |  | 1 | |
| colin30 | 150 | 165 | 167 | 179 | 189 | 189 | 174 | 180 | 158 | 166 | 150 | 150 | 165 | 169 |  | 1 | |
| ferna1 | 153 | 153 | 167 | 179 | 186 | 186 | 174 | 180 | 166 | 170 | 142 | 150 | 165 | 169 |  | 1 | |
| ferna2 | 153 | 153 | 167 | 179 | 186 | 186 | 174 | 180 | 166 | 170 | 150 | 150 | 165 | 169 |  | 1 | |
| ferna3 | 150 | 165 | 176 | 179 | 186 | 189 | 174 | 180 | 160 | 166 | 150 | 150 | 165 | 169 |  | 1 | |
| ferna4 | 153 | 153 | 167 | 179 | 177 | 189 | 177 | 180 | 160 | 162 | 150 | 150 | 165 | 169 |  | 1 | |
| ferna5 | 153 | 153 | 167 | 179 | 186 | 189 | 174 | 180 | 166 | 170 | 142 | 164 | 165 | 169 |  | 1 | |
| ferna6 | 153 | 153 | 167 | 179 | 177 | 189 | 177 | 180 | 160 | 166 | 150 | 150 | 165 | 169 |  | 1 | |
| ferna7 | 150 | 165 | 167 | 179 | 189 | 189 | 180 | 180 | 160 | 162 | 142 | 150 | 165 | 169 |  | 1 | |
| ferna8 | 153 | 153 | 176 | 179 | 186 | 186 | 174 | 180 | 166 | 170 | 142 | 150 | 165 | 169 |  | 1 | |
| ferna9 | 153 | 153 | 167 | 179 | 177 | 189 | 174 | 180 | 160 | 166 | 150 | 150 | 165 | 169 |  | 1 | |
| ferna10 | 150 | 165 | 167 | 179 | 186 | 189 | 174 | 180 | 160 | 162 | 144 | 144 | 165 | 169 |  | 1 | |
| ferna11 | 150 | 165 | 167 | 179 | 186 | 186 | 174 | 180 | 160 | 166 | 142 | 150 | 165 | 169 |  | 1 | |
| ferna12 | 150 | 165 | 167 | 179 | 186 | 186 | 177 | 180 | 160 | 162 | 142 | 150 | 169 | 171 |  | 1 | |
| ferna13 | 153 | 165 | 167 | 179 | 186 | 186 | 177 | 180 | 160 | 162 | 142 | 150 | 165 | 169 |  | 1 | |
| ferna14 | 153 | 165 | 167 | 179 | 186 | 186 | 177 | 180 | 160 | 166 | 142 | 150 | 165 | 173 |  | 1 | |
| ferna15 | 150 | 150 | 170 | 176 | 186 | 186 | 174 | 180 | 166 | 170 | 150 | 150 | 165 | 173 |  | 1 | |
| ferna16 | 150 | 150 | 167 | 179 | 186 | 186 | 174 | 180 | 166 | 170 | 150 | 150 | 165 | 173 |  | 1 | |
| ferna17 | 150 | 165 | 167 | 179 | 186 | 186 | 174 | 180 | 160 | 166 | 142 | 142 | 169 | 179 |  | 1 | |
| ferna18 | 153 | 165 | 167 | 179 | 177 | 189 | 177 | 180 | 156 | 162 | 142 | 150 | 169 | 179 |  | 1 | |
| ferna19 | 153 | 165 | 167 | 179 | 186 | 189 | 177 | 180 | 160 | 166 | 150 | 164 | 169 | 179 |  | 1 | |
| ferna20 | 150 | 165 | 167 | 179 | 177 | 189 | 174 | 180 | 160 | 162 | 142 | 150 | 171 | 173 |  | 1 | |
| ferna21 | 150 | 165 | 167 | 179 | 177 | 189 | 177 | 180 | 160 | 162 | 142 | 150 | 171 | 173 |  | 1 | |
| ferna22 | 150 | 150 | 167 | 179 | 186 | 186 | 177 | 180 | 160 | 170 | 142 | 150 | 171 | 173 |  | 1 | |
| ferna23 | 153 | 165 | 167 | 179 | 177 | 189 | 177 | 180 | 160 | 162 | 142 | 150 | 171 | 173 |  | 1 | |
| ferna24 | 153 | 165 | 167 | 179 | 186 | 189 | 177 | 180 | 160 | 162 | 142 | 150 | 171 | 173 |  | 1 | |
| ferna25 | 150 | 165 | 167 | 179 | 186 | 189 | 177 | 180 | 160 | 162 | 142 | 150 | 171 | 173 |  | 1 | |
| ferna26 | 150 | 153 | 167 | 179 | 177 | 177 | 174 | 180 | 160 | 166 | 142 | 150 | 171 | 173 |  | 1 | |
| ferna27 | 150 | 165 | 167 | 179 | 186 | 189 | 174 | 180 | 160 | 162 | 142 | 150 | 165 | 165 |  | 1 | |
| ferna28 | 150 | 150 | 176 | 179 | 177 | 189 | 174 | 180 | 160 | 166 | 142 | 150 | 165 | 165 |  | 1 | |
| ferna29 | 150 | 165 | 176 | 179 | 186 | 189 | 174 | 180 | 160 | 162 | 142 | 150 | 169 | 173 |  | 1 | |
| ancoa1 | 153 | 153 | 176 | 179 | 186 | 186 | 177 | 180 | 160 | 162 | 144 | 164 | 165 | 165 |  | 1 | |
| ancoa2 | 153 | 153 | 176 | 179 | 186 | 186 | 177 | 180 | 160 | 162 | 144 | 150 | 169 | 173 |  | 1 | |
| ancoa3 | 153 | 165 | 176 | 179 | 186 | 186 | 177 | 180 | 160 | 170 | 142 | 146 | 169 | 173 |  | 1 | |
| ancoa4 | 153 | 153 | 167 | 167 | 186 | 189 | 174 | 180 | 162 | 172 | 150 | 164 | 165 | 169 |  | 1 | |
| ancoa5 | 150 | 153 | 176 | 179 | 186 | 189 | 174 | 180 | 156 | 160 | 150 | 164 | 169 | 171 |  | 1 | |
| ancoa6 | 150 | 150 | 176 | 179 | 186 | 189 | 174 | 180 | 156 | 160 | 148 | 164 | 169 | 173 |  | 1 | |
| ancoa7 | 153 | 153 | 176 | 179 | 186 | 189 | 174 | 180 | 156 | 160 | 148 | 164 | 169 | 173 |  | 1 | |
| ancoa8 | 153 | 153 | 176 | 179 | 186 | 189 | 174 | 180 | 162 | 170 | 146 | 150 | 169 | 171 |  | 1 | |
| ancoa9 | 150 | 150 | 170 | 176 | 186 | 189 | 174 | 180 | 162 | 170 | 150 | 164 | 169 | 173 |  | 1 | |
| ancoa10 | 150 | 153 | 170 | 176 | 186 | 189 | 174 | 180 | 162 | 170 | 150 | 164 | 169 | 171 |  | 1 | |
| ancoa11 | 153 | 153 | 176 | 179 | 186 | 189 | 177 | 180 | 162 | 170 | 148 | 160 | 169 | 171 |  | 1 | |
| ancoa12 | 153 | 153 | 176 | 179 | 186 | 189 | 174 | 180 | 156 | 160 | 148 | 160 | 169 | 173 |  | 1 | |
| ancoa13 | 153 | 153 | 176 | 179 | 186 | 189 | 177 | 180 | 156 | 160 | 148 | 160 | 169 | 173 |  | 1 | |
| ancoa14 | 150 | 150 | 170 | 176 | 186 | 189 | 177 | 180 | 160 | 170 | 150 | 164 | 165 | 173 |  | 1 | |
| ancoa15 | 150 | 150 | 176 | 179 | 189 | 189 | 177 | 180 | 162 | 170 | 150 | 164 | 165 | 173 |  | 1 | |
| ancoa16 | 153 | 153 | 176 | 179 | 186 | 189 | 177 | 180 | 162 | 170 | 148 | 164 | 169 | 171 |  | 1 | |
| ancoa17 | 153 | 153 | 176 | 179 | 186 | 189 | 177 | 180 | 156 | 170 | 148 | 164 | 165 | 173 |  | 1 | |
| ancoa18 | 153 | 153 | 176 | 179 | 186 | 189 | 177 | 180 | 156 | 170 | 148 | 164 | 169 | 173 |  | 1 | |
| ancoa19 | 150 | 153 | 176 | 179 | 186 | 189 | 177 | 180 | 156 | 170 | 148 | 164 | 169 | 173 |  | 1 | |
| ancoa20 | 153 | 153 | 176 | 179 | 186 | 189 | 174 | 180 | 156 | 170 | 148 | 164 | 173 | 173 |  | 1 | |
| ancoa21 | 150 | 150 | 176 | 179 | 186 | 189 | 177 | 180 | 162 | 170 | 150 | 164 | 169 | 173 |  | 1 | |
| ancoa23 | 150 | 153 | 176 | 179 | 177 | 186 | 177 | 180 | 162 | 170 | 150 | 164 | 169 | 173 |  | 1 | |
| ancoa24 | 150 | 153 | 176 | 179 | 186 | 189 | 177 | 180 | 162 | 170 | 150 | 164 | 169 | 173 |  | 2 | |
| ancoa25 | 165 | 165 | 170 | 179 | 186 | 189 | 177 | 180 | 160 | 166 | 150 | 164 | 169 | 173 |  | 1 | |
| ancoa26 | 150 | 153 | 176 | 179 | 186 | 189 | 177 | 180 | 156 | 162 | 150 | 164 | 169 | 173 |  | 1 | |
| ancoa27 | 153 | 153 | 176 | 179 | 186 | 189 | 177 | 180 | 162 | 170 | 150 | 164 | 169 | 171 |  | 1 | |
| ancoa28 | 150 | 153 | 176 | 179 | 186 | 189 | 160 | 177 | 160 | 170 | 150 | 164 | 169 | 171 |  | 1 | |
| ancoa29 | 150 | 150 | 176 | 179 | 186 | 189 | 177 | 180 | 160 | 170 | 134 | 150 | 169 | 171 |  | 1 | |
| ancoa30 | 150 | 150 | 176 | 179 | 177 | 186 | 177 | 180 | 160 | 170 | 150 | 164 | 169 | 171 |  | 1 | |
| ancoa31 | 163 | 163 | 176 | 179 | 186 | 189 | 174 | 180 | 160 | 166 | 146 | 164 | 165 | 173 |  | 1 | |
| ancoa32 | 165 | 165 | 176 | 179 | 186 | 189 | 174 | 180 | 166 | 170 | 134 | 150 | 165 | 173 |  | 1 | |
| ancoa33 | 153 | 153 | 176 | 179 | 186 | 189 | 174 | 180 | 156 | 166 | 148 | 164 | 165 | 173 |  | 1 | |
| ancoa34 | 153 | 153 | 176 | 179 | 177 | 189 | 174 | 180 | 160 | 166 | 150 | 164 | 165 | 169 |  | 1 | |
| ancoa35 | 153 | 153 | 176 | 179 | 186 | 189 | 177 | 180 | 160 | 160 | 142 | 150 | 165 | 173 |  | 1 | |
| ancoa36 | 153 | 153 | 176 | 179 | 186 | 189 | 177 | 180 | 160 | 170 | 142 | 150 | 165 | 173 |  | 1 | |
| ancoa37 | 153 | 165 | 176 | 179 | 186 | 189 | 177 | 180 | 160 | 170 | 134 | 150 | 165 | 173 |  | 1 | |
| alema1 | 150 | 165 | 176 | 179 | 186 | 186 | 174 | 180 | 158 | 166 | 148 | 164 | 169 | 171 |  | 1 | |
| alema2 | 150 | 150 | 176 | 179 | 186 | 186 | 174 | 180 | 158 | 158 | 144 | 164 | 173 | 173 |  | 1 | |
| alema3 | 150 | 150 | 176 | 179 | 186 | 189 | 174 | 180 | 158 | 166 | 150 | 164 | 165 | 173 |  | 1 | |
| alema4 | 150 | 150 | 176 | 179 | 177 | 189 | 174 | 180 | 160 | 170 | 150 | 154 | 165 | 173 |  | 1 | |
| alema5 | 150 | 150 | 176 | 179 | 186 | 189 | 174 | 180 | 160 | 170 | 142 | 150 | 165 | 169 |  | 1 | |
| alema6 | 150 | 150 | 176 | 179 | 189 | 189 | 174 | 180 | 160 | 170 | 142 | 150 | 165 | 169 |  | 1 | |
| alema7 | 150 | 150 | 176 | 179 | 177 | 186 | 174 | 180 | 160 | 170 | 142 | 150 | 165 | 169 |  | 1 | |
| alema8 | 150 | 150 | 176 | 179 | 177 | 186 | 174 | 180 | 158 | 166 | 142 | 150 | 169 | 169 |  | 1 | |
| alema9 | 150 | 150 | 176 | 179 | 177 | 186 | 174 | 180 | 162 | 166 | 150 | 164 | 169 | 169 |  | 1 | |
| alema10 | 150 | 153 | 176 | 179 | 177 | 186 | 174 | 180 | 158 | 166 | 150 | 164 | 169 | 169 |  | 1 | |
| alema11 | 150 | 165 | 176 | 179 | 186 | 189 | 174 | 180 | 162 | 166 | 150 | 164 | 169 | 169 |  | 1 | |
| alema12 | 153 | 165 | 176 | 179 | 186 | 189 | 174 | 180 | 162 | 170 | 150 | 164 | 169 | 169 |  | 1 | |
| alema13 | 150 | 165 | 176 | 179 | 186 | 189 | 174 | 180 | 158 | 158 | 138 | 144 | 169 | 169 |  | 1 | |
| alema14 | 150 | 150 | 176 | 179 | 186 | 189 | 174 | 180 | 158 | 158 | 138 | 144 | 165 | 173 |  | 1 | |
| alema15 | 150 | 153 | 167 | 170 | 177 | 189 | 174 | 180 | 160 | 170 | 142 | 150 | 165 | 173 |  | 1 | |
| alema16 | 150 | 153 | 176 | 179 | 177 | 186 | 174 | 180 | 160 | 170 | 142 | 150 | 173 | 173 |  | 1 | |
| alema17 | 153 | 165 | 167 | 170 | 186 | 189 | 174 | 180 | 158 | 166 | 144 | 144 | 173 | 173 |  | 1 | |
| alema18 | 150 | 156 | 176 | 179 | 186 | 189 | 174 | 180 | 160 | 170 | 150 | 150 | 165 | 173 |  | 1 | |
| alema20 | 150 | 153 | 167 | 170 | 186 | 189 | 174 | 180 | 160 | 170 | 142 | 150 | 165 | 173 |  | 1 | |
| alema21 | 150 | 153 | 176 | 179 | 186 | 189 | 174 | 180 | 160 | 170 | 142 | 150 | 165 | 171 |  | 1 | |
| alema22 | 150 | 153 | 176 | 179 | 177 | 186 | 177 | 180 | 160 | 170 | 142 | 150 | 165 | 171 |  | 1 | |
| alema23 | 153 | 153 | 176 | 179 | 186 | 186 | 174 | 180 | 160 | 170 | 142 | 150 | 165 | 169 |  | 1 | |
| alema24 | 153 | 156 | 176 | 179 | 186 | 189 | 174 | 180 | 160 | 170 | 150 | 164 | 165 | 173 |  | 1 | |
| alema25 | 153 | 156 | 176 | 179 | 186 | 189 | 174 | 180 | 160 | 170 | 142 | 150 | 165 | 173 |  | 1 | |
| alema27 | 150 | 153 | 176 | 179 | 186 | 189 | 174 | 180 | 160 | 170 | 142 | 150 | 165 | 173 |  | 2 | |
| alema28 | 150 | 153 | 176 | 179 | 186 | 189 | 174 | 180 | 158 | 166 | 142 | 150 | 159 | 169 |  | 1 | |
| alema29 | 150 | 153 | 176 | 179 | 186 | 189 | 174 | 180 | 160 | 170 | 150 | 164 | 169 | 173 |  | 1 | |
| rastr1 | 153 | 156 | 176 | 179 | 186 | 189 | 177 | 180 | 164 | 166 | 142 | 150 | 165 | 169 |  | 1 | |
| rastr2 | 153 | 156 | 176 | 179 | 180 | 189 | 174 | 180 | 164 | 166 | 150 | 150 | 165 | 173 |  | 1 | |
| rastr3 | 153 | 156 | 176 | 179 | 180 | 189 | 174 | 180 | 160 | 166 | 150 | 150 | 165 | 173 |  | 1 | |
| rastr4 | 153 | 156 | 176 | 179 | 180 | 189 | 174 | 180 | 160 | 166 | 142 | 150 | 165 | 173 |  | 1 | |
| rastr5 | 153 | 165 | 176 | 179 | 180 | 189 | 174 | 180 | 164 | 166 | 150 | 150 | 165 | 173 |  | 1 | |
| rastr6 | 150 | 153 | 176 | 179 | 180 | 189 | 174 | 180 | 160 | 166 | 142 | 150 | 165 | 173 |  | 1 | |
| rastr7 | 150 | 153 | 176 | 179 | 180 | 189 | 174 | 180 | 160 | 170 | 134 | 150 | 165 | 173 |  | 1 | |
| rastr8 | 153 | 165 | 176 | 179 | 180 | 189 | 174 | 180 | 164 | 166 | 134 | 178 | 169 | 169 |  | 1 | |
| rastr9 | 153 | 165 | 176 | 179 | 189 | 189 | 174 | 180 | 166 | 170 | 128 | 134 | 169 | 169 |  | 1 | |
| rastr10 | 150 | 156 | 167 | 179 | 180 | 189 | 177 | 180 | 164 | 166 | 128 | 134 | 165 | 173 |  | 1 | |
| rastr11 | 150 | 156 | 176 | 179 | 180 | 189 | 174 | 180 | 160 | 166 | 122 | 132 | 169 | 169 |  | 1 | |
| rastr12 | 150 | 156 | 167 | 179 | 186 | 186 | 177 | 180 | 164 | 166 | 144 | 160 | 165 | 173 |  | 1 | |
| rastr13 | 156 | 165 | 176 | 179 | 186 | 186 | 174 | 180 | 170 | 170 | 144 | 150 | 165 | 173 |  | 1 | |
| rastr14 | 153 | 165 | 176 | 179 | 180 | 189 | 174 | 180 | 164 | 166 | 138 | 150 | 165 | 169 |  | 1 | |
| rastr16 | 156 | 156 | 167 | 168 | 180 | 189 | 174 | 180 | 162 | 170 | 144 | 150 | 165 | 173 |  | 1 | |
| rastr17 | 153 | 165 | 176 | 179 | 180 | 189 | 174 | 180 | 160 | 160 | 142 | 142 | 165 | 169 |  | 1 | |
| rastr19 | 153 | 165 | 176 | 179 | 180 | 189 | 174 | 180 | 164 | 166 | 144 | 150 | 165 | 173 |  | 3 | |
| rastr20 | 153 | 165 | 176 | 179 | 180 | 189 | 174 | 180 | 160 | 170 | 144 | 150 | 169 | 169 |  | 1 | |
| rastr21 | 153 | 165 | 176 | 179 | 186 | 189 | 174 | 180 | 164 | 166 | 144 | 150 | 165 | 173 |  | 1 | |
| rastr22 | 153 | 165 | 176 | 179 | 177 | 186 | 174 | 180 | 164 | 166 | 144 | 150 | 165 | 173 |  | 1 | |
| rastr24 | 153 | 165 | 176 | 179 | 180 | 180 | 174 | 180 | 166 | 166 | 142 | 150 | 165 | 173 |  | 1 | |
| rastr25 | 153 | 165 | 167 | 179 | 177 | 186 | 174 | 180 | 160 | 170 | 144 | 150 | 169 | 169 |  | 1 | |
| rastr26 | 150 | 150 | 176 | 179 | 177 | 189 | 174 | 180 | 160 | 170 | 142 | 150 | 165 | 165 |  | 1 | |
| rastr27 | 153 | 165 | 176 | 179 | 180 | 189 | 174 | 180 | 160 | 166 | 144 | 150 | 165 | 173 |  | 2 | |
| rastr28 | 153 | 165 | 176 | 179 | 189 | 189 | 174 | 180 | 160 | 170 | 150 | 150 | 165 | 169 |  | 1 | |
| rastr29 | 150 | 153 | 176 | 179 | 180 | 189 | 174 | 180 | 160 | 170 | 144 | 150 | 165 | 169 |  | 1 | |
| rastr30 | 150 | 165 | 176 | 179 | 186 | 189 | 174 | 180 | 160 | 166 | 150 | 164 | 169 | 169 |  | 1 | |
| maite1 | 150 | 150 | 176 | 179 | 186 | 189 | 171 | 180 | 160 | 166 | 144 | 150 | 173 | 269 |  | 1 | |
| maite2 | 153 | 153 | 176 | 179 | 186 | 189 | 177 | 180 | 160 | 166 | 138 | 142 | 165 | 169 |  | 1 | |
| maite3 | 150 | 150 | 176 | 179 | 186 | 189 | 171 | 180 | 160 | 166 | 150 | 164 | 165 | 169 |  | 1 | |
| maite4 | 153 | 165 | 176 | 179 | 186 | 189 | 174 | 180 | 160 | 170 | 142 | 150 | 169 | 173 |  | 1 | |
| maite5 | 153 | 165 | 176 | 179 | 189 | 189 | 174 | 180 | 164 | 166 | 138 | 142 | 165 | 169 |  | 1 | |
| maite6 | 150 | 150 | 176 | 179 | 189 | 189 | 174 | 180 | 160 | 166 | 138 | 142 | 165 | 173 |  | 1 | |
| maite7 | 150 | 150 | 176 | 179 | 186 | 189 | 174 | 180 | 160 | 160 | 138 | 142 | 169 | 173 |  | 1 | |
| maite8 | 150 | 153 | 176 | 179 | 186 | 189 | 174 | 180 | 160 | 170 | 142 | 150 | 165 | 169 |  | 3 | |
| maite9 | 153 | 165 | 176 | 179 | 189 | 189 | 174 | 180 | 160 | 170 | 142 | 150 | 171 | 173 |  | 1 | |
| maite10 | 153 | 165 | 176 | 179 | 189 | 189 | 174 | 180 | 160 | 170 | 142 | 150 | 165 | 173 |  | 1 | |
| maite11 | 150 | 150 | 176 | 179 | 186 | 189 | 177 | 180 | 156 | 162 | 144 | 150 | 169 | 173 |  | 1 | |
| maite12 | 153 | 165 | 176 | 179 | 189 | 189 | 174 | 180 | 162 | 166 | 144 | 150 | 165 | 169 |  | 1 | |
| maite13 | 153 | 165 | 176 | 179 | 186 | 189 | 174 | 180 | 162 | 170 | 150 | 164 | 165 | 169 |  | 1 | |
| maite14 | 153 | 165 | 176 | 179 | 189 | 189 | 174 | 180 | 164 | 166 | 142 | 150 | 165 | 169 |  | 1 | |
| maite15 | 153 | 165 | 176 | 179 | 186 | 189 | 174 | 180 | 164 | 166 | 142 | 164 | 169 | 169 |  | 1 | |
| maite16 | 165 | 165 | 176 | 179 | 189 | 189 | 177 | 180 | 160 | 166 | 142 | 164 | 171 | 171 |  | 1 | |
| maite17 | 150 | 150 | 176 | 179 | 186 | 189 | 177 | 180 | 166 | 166 | 142 | 164 | 165 | 169 |  | 1 | |
| maite18 | 150 | 153 | 176 | 179 | 186 | 189 | 177 | 180 | 160 | 166 | 142 | 150 | 165 | 169 |  | 1 | |
| maite20 | 150 | 150 | 176 | 179 | 186 | 189 | 177 | 180 | 166 | 170 | 142 | 164 | 169 | 169 |  | 1 | |
| maite21 | 153 | 165 | 167 | 179 | 186 | 189 | 174 | 180 | 160 | 170 | 142 | 150 | 169 | 173 |  | 1 | |
| maite22 | 150 | 153 | 176 | 179 | 186 | 189 | 177 | 180 | 160 | 166 | 142 | 150 | 169 | 173 |  | 1 | |
| maite23 | 150 | 150 | 176 | 179 | 186 | 189 | 174 | 180 | 160 | 166 | 142 | 150 | 169 | 173 |  | 1 | |
| maite24 | 150 | 153 | 176 | 179 | 186 | 189 | 174 | 180 | 166 | 179 | 142 | 150 | 165 | 169 |  | 1 | |
| maite25 | 165 | 165 | 176 | 179 | 186 | 189 | 177 | 180 | 162 | 166 | 142 | 150 | 169 | 173 |  | 1 | |
| maite26 | 150 | 153 | 169 | 179 | 189 | 195 | 177 | 180 | 160 | 162 | 150 | 164 | 169 | 173 |  | 1 | |
| maite27 | 153 | 153 | 176 | 179 | 189 | 189 | 177 | 180 | 160 | 170 | 144 | 150 | 165 | 169 |  | 1 | |
| maite28 | 150 | 150 | 176 | 179 | 186 | 189 | 174 | 180 | 160 | 166 | 138 | 142 | 169 | 173 |  | 1 | |
| maite29 | 150 | 153 | 176 | 179 | 186 | 189 | 177 | 180 | 160 | 170 | 150 | 164 | 171 | 173 |  | 1 | |
| maite30 | 150 | 150 | 176 | 179 | 186 | 189 | 174 | 180 | 160 | 166 | 138 | 142 | 171 | 173 |  | 1 | |
| maite31 | 150 | 150 | 176 | 179 | 186 | 189 | 171 | 180 | 160 | 166 | 134 | 150 | 169 | 173 |  | 1 | |
| maite32 | 153 | 165 | 176 | 179 | 186 | 189 | 171 | 180 | 160 | 170 | 128 | 142 | 171 | 173 |  | 1 | |
| maite33 | 150 | 150 | 176 | 179 | 186 | 189 | 174 | 180 | 156 | 160 | 134 | 150 | 165 | 169 |  | 1 | |
| maite35 | 153 | 153 | 176 | 179 | 186 | 189 | 174 | 180 | 156 | 166 | 134 | 150 | 171 | 173 |  | 1 | |
| maite36 | 153 | 153 | 176 | 179 | 186 | 186 | 177 | 180 | 160 | 166 | 134 | 150 | 165 | 169 |  | 1 | |
| maite37 | 153 | 153 | 176 | 179 | 186 | 186 | 177 | 180 | 160 | 170 | 134 | 150 | 165 | 169 |  | 1 | |
| maite38 | 150 | 150 | 176 | 179 | 186 | 186 | 171 | 180 | 156 | 170 | 128 | 142 | 169 | 173 |  | 1 | |
| maite40 | 150 | 150 | 176 | 179 | 186 | 189 | 174 | 180 | 156 | 162 | 134 | 150 | 171 | 173 |  | 1 | |
| maite41 | 150 | 150 | 176 | 179 | 186 | 189 | 171 | 180 | 156 | 162 | 128 | 142 | 165 | 169 |  | 1 | |
| maite42 | 150 | 150 | 176 | 179 | 186 | 189 | 171 | 180 | 160 | 166 | 128 | 142 | 165 | 169 |  | 1 | |
| maite43 | 153 | 165 | 176 | 179 | 186 | 189 | 171 | 180 | 160 | 166 | 128 | 142 | 165 | 169 |  | 3 | |
| maite45 | 153 | 165 | 176 | 179 | 186 | 189 | 174 | 180 | 156 | 160 | 134 | 150 | 171 | 173 |  | 1 | |
| maite46 | 153 | 165 | 176 | 179 | 186 | 189 | 174 | 180 | 160 | 170 | 134 | 150 | 171 | 173 |  | 1 | |
| maite47 | 153 | 165 | 176 | 179 | 186 | 189 | 174 | 180 | 160 | 166 | 134 | 150 | 171 | 173 |  | 2 | |
| maite48 | 153 | 165 | 176 | 179 | 186 | 189 | 174 | 180 | 160 | 160 | 134 | 150 | 171 | 173 |  | 1 | |
| maite49 | 153 | 153 | 176 | 179 | 186 | 189 | 177 | 180 | 160 | 170 | 134 | 150 | 165 | 169 |  | 1 | |
| maite50 | 153 | 165 | 176 | 179 | 177 | 186 | 174 | 180 | 160 | 160 | 134 | 150 | 169 | 173 |  | 1 | |
| maite51 | 153 | 153 | 176 | 179 | 186 | 189 | 177 | 180 | 162 | 166 | 134 | 150 | 171 | 173 |  | 1 | |
| maite52 | 153 | 153 | 176 | 179 | 186 | 189 | 177 | 180 | 160 | 170 | 150 | 164 | 165 | 169 |  | 1 | |
| maite53 | 153 | 165 | 176 | 179 | 186 | 189 | 174 | 180 | 156 | 166 | 150 | 150 | 169 | 173 |  | 1 | |
| maite54 | 153 | 153 | 176 | 179 | 186 | 189 | 174 | 180 | 162 | 166 | 142 | 150 | 171 | 173 |  | 1 | |
| maite55 | 150 | 150 | 170 | 176 | 186 | 189 | 174 | 180 | 156 | 166 | 134 | 150 | 169 | 173 |  | 1 | |
| maite56 | 150 | 150 | 176 | 179 | 186 | 186 | 171 | 180 | 156 | 166 | 134 | 150 | 165 | 173 |  | 1 | |
| maite57 | 153 | 153 | 176 | 179 | 186 | 186 | 177 | 180 | 156 | 166 | 134 | 150 | 169 | 173 |  | 1 | |
| maite58 | 153 | 153 | 170 | 176 | 186 | 186 | 174 | 180 | 156 | 166 | 134 | 150 | 165 | 169 |  | 1 | |
| miraf1 | 150 | 150 | 176 | 179 | 180 | 189 | 171 | 178 | 160 | 166 | 144 | 150 | 169 | 173 |  | 1 | |
| miraf4 | 153 | 153 | 176 | 179 | 180 | 189 | 171 | 178 | 160 | 162 | 144 | 150 | 169 | 173 |  | 3 | |
| miraf5 | 153 | 153 | 176 | 179 | 177 | 189 | 177 | 180 | 160 | 162 | 144 | 150 | 169 | 173 |  | 1 | |
| miraf6 | 150 | 150 | 167 | 179 | 186 | 186 | 177 | 180 | 160 | 162 | 144 | 150 | 169 | 173 |  | 1 | |
| miraf7 | 153 | 153 | 176 | 179 | 186 | 186 | 171 | 178 | 160 | 162 | 144 | 150 | 169 | 173 |  | 1 | |
| miraf8 | 153 | 153 | 176 | 179 | 186 | 186 | 171 | 178 | 160 | 166 | 144 | 150 | 169 | 173 |  | 1 | |
| miraf9 | 150 | 150 | 167 | 179 | 186 | 186 | 171 | 178 | 160 | 162 | 144 | 150 | 169 | 173 |  | 1 | |
| miraf10 | 150 | 150 | 176 | 179 | 180 | 186 | 174 | 180 | 160 | 162 | 144 | 150 | 169 | 173 |  | 1 | |
| miraf11 | 153 | 153 | 176 | 179 | 180 | 189 | 171 | 178 | 160 | 166 | 144 | 150 | 169 | 173 |  | 1 | |
| miraf12 | 153 | 153 | 176 | 179 | 186 | 189 | 171 | 178 | 160 | 170 | 144 | 150 | 169 | 169 |  | 1 | |
| miraf13 | 153 | 153 | 176 | 179 | 186 | 189 | 171 | 178 | 158 | 170 | 144 | 150 | 169 | 169 |  | 1 | |
| miraf14 | 150 | 150 | 167 | 179 | 186 | 189 | 177 | 180 | 160 | 170 | 144 | 150 | 169 | 173 |  | 1 | |
| miraf15 | 153 | 153 | 176 | 179 | 180 | 189 | 171 | 178 | 158 | 162 | 144 | 150 | 169 | 169 |  | 1 | |
| miraf16 | 153 | 153 | 167 | 179 | 186 | 189 | 177 | 180 | 160 | 170 | 144 | 150 | 169 | 173 |  | 1 | |
| miraf17 | 150 | 153 | 167 | 179 | 186 | 186 | 177 | 180 | 158 | 160 | 144 | 150 | 169 | 173 |  | 1 | |
| miraf18 | 153 | 153 | 176 | 179 | 180 | 189 | 174 | 180 | 158 | 160 | 144 | 150 | 169 | 173 |  | 1 | |
| miraf19 | 153 | 153 | 176 | 179 | 180 | 189 | 174 | 180 | 158 | 160 | 144 | 150 | 165 | 169 |  | 1 | |
| miraf20 | 153 | 153 | 176 | 179 | 180 | 189 | 174 | 180 | 160 | 166 | 144 | 150 | 169 | 173 |  | 1 | |
| miraf21 | 160 | 160 | 176 | 179 | 186 | 189 | 174 | 180 | 160 | 162 | 150 | 164 | 169 | 173 |  | 1 | |
| miraf22 | 150 | 165 | 176 | 179 | 177 | 189 | 174 | 180 | 156 | 162 | 128 | 150 | 169 | 173 |  | 1 | |
| miraf23 | 153 | 153 | 176 | 179 | 177 | 189 | 177 | 180 | 156 | 162 | 128 | 150 | 169 | 173 |  | 1 | |
| miraf24 | 150 | 165 | 167 | 179 | 186 | 189 | 171 | 177 | 160 | 166 | 128 | 150 | 169 | 173 |  | 1 | |
| miraf25 | 150 | 150 | 167 | 179 | 177 | 186 | 174 | 180 | 160 | 166 | 150 | 150 | 171 | 173 |  | 1 | |
| miraf26 | 150 | 150 | 176 | 179 | 177 | 189 | 180 | 180 | 160 | 170 | 128 | 164 | 169 | 171 |  | 1 | |
| miraf27 | 153 | 153 | 176 | 179 | 177 | 189 | 171 | 177 | 158 | 162 | 150 | 164 | 171 | 173 |  | 1 | |
| miraf28 | 150 | 150 | 167 | 174 | 186 | 186 | 171 | 177 | 160 | 166 | 150 | 150 | 171 | 173 |  | 1 | |
| miraf29 | 153 | 153 | 176 | 179 | 177 | 189 | 174 | 180 | 158 | 162 | 128 | 150 | 169 | 173 |  | 1 | |
| miraf30 | 150 | 150 | 167 | 179 | 186 | 186 | 174 | 180 | 160 | 166 | 150 | 164 | 171 | 173 |  | 1 | |
| lomas1 | 150 | 150 | 170 | 179 | 186 | 186 | 174 | 180 | 160 | 166 | 134 | 150 | 171 | 173 |  | 1 | |
| lomas2 | 150 | 165 | 176 | 179 | 186 | 186 | 174 | 180 | 160 | 166 | 144 | 150 | 169 | 173 |  | 1 | |
| lomas3 | 150 | 150 | 176 | 179 | 189 | 189 | 174 | 180 | 160 | 166 | 150 | 164 | 169 | 173 |  | 1 | |
| lomas4 | 150 | 150 | 167 | 179 | 177 | 186 | 171 | 180 | 160 | 166 | 134 | 150 | 169 | 171 |  | 1 | |
| lomas5 | 153 | 153 | 176 | 179 | 177 | 189 | 171 | 180 | 158 | 160 | 150 | 164 | 169 | 171 |  | 1 | |
| lomas6 | 150 | 150 | 167 | 179 | 177 | 186 | 177 | 180 | 158 | 160 | 144 | 150 | 169 | 173 |  | 1 | |
| lomas7 | 153 | 153 | 175 | 179 | 189 | 189 | 177 | 180 | 162 | 166 | 144 | 150 | 169 | 173 |  | 1 | |
| lomas8 | 150 | 165 | 176 | 179 | 186 | 189 | 171 | 177 | 162 | 166 | 144 | 150 | 169 | 173 |  | 1 | |
| lomas9 | 150 | 150 | 170 | 179 | 186 | 189 | 171 | 177 | 160 | 166 | 150 | 164 | 169 | 173 |  | 1 | |
| lomas10 | 153 | 165 | 176 | 179 | 189 | 189 | 171 | 177 | 160 | 166 | 150 | 164 | 171 | 171 |  | 1 | |
| lomas11 | 150 | 150 | 176 | 179 | 186 | 186 | 171 | 177 | 162 | 166 | 144 | 150 | 165 | 171 |  | 1 | |
| lomas12 | 153 | 153 | 176 | 179 | 186 | 186 | 171 | 177 | 162 | 166 | 150 | 164 | 171 | 173 |  | 1 | |
| lomas13 | 150 | 165 | 176 | 179 | 186 | 186 | 177 | 180 | 160 | 162 | 142 | 150 | 171 | 173 |  | 1 | |
| lomas14 | 150 | 165 | 167 | 179 | 189 | 189 | 171 | 177 | 156 | 160 | 134 | 150 | 165 | 173 |  | 1 | |
| lomas15 | 150 | 150 | 176 | 179 | 186 | 189 | 171 | 177 | 156 | 160 | 142 | 150 | 173 | 173 |  | 1 | |
| lomas16 | 150 | 165 | 176 | 179 | 186 | 189 | 174 | 180 | 156 | 160 | 150 | 164 | 171 | 173 |  | 1 | |
| lomas17 | 150 | 150 | 176 | 179 | 189 | 189 | 171 | 180 | 156 | 160 | 142 | 150 | 171 | 173 |  | 1 | |
| lomas18 | 153 | 153 | 170 | 179 | 186 | 189 | 171 | 180 | 160 | 166 | 142 | 150 | 171 | 173 |  | 1 | |
| lomas19 | 153 | 153 | 170 | 179 | 186 | 189 | 171 | 177 | 156 | 160 | 134 | 150 | 169 | 173 |  | 1 | |
| lomas20 | 150 | 150 | 176 | 179 | 189 | 189 | 171 | 177 | 162 | 166 | 142 | 150 | 169 | 173 |  | 1 | |
| lomas21 | 153 | 153 | 176 | 179 | 186 | 189 | 171 | 180 | 160 | 166 | 142 | 150 | 169 | 171 |  | 1 | |
| lomas22 | 150 | 150 | 167 | 179 | 186 | 189 | 171 | 180 | 160 | 166 | 134 | 150 | 173 | 173 |  | 1 | |
| lomas23 | 150 | 150 | 176 | 179 | 186 | 189 | 171 | 180 | 160 | 166 | 128 | 142 | 171 | 173 |  | 1 | |
| lomas24 | 150 | 150 | 176 | 179 | 186 | 189 | 171 | 180 | 160 | 166 | 128 | 142 | 165 | 173 |  | 1 | |
| lomas25 | 150 | 153 | 176 | 179 | 189 | 189 | 177 | 177 | 156 | 160 | 128 | 142 | 169 | 173 |  | 1 | |
| lomas26 | 150 | 150 | 176 | 179 | 189 | 189 | 177 | 177 | 162 | 166 | 142 | 150 | 169 | 169 |  | 1 | |
| lomas27 | 153 | 153 | 176 | 179 | 186 | 186 | 177 | 180 | 160 | 166 | 142 | 150 | 169 | 169 |  | 1 | |
| niche1 | 150 | 165 | 176 | 179 | 186 | 189 | 177 | 180 | 160 | 166 | 142 | 150 | 169 | 169 |  | 1 | |
| niche2 | 150 | 150 | 176 | 179 | 186 | 189 | 180 | 180 | 156 | 166 | 134 | 150 | 169 | 169 |  | 1 | |
| niche3 | 150 | 150 | 167 | 179 | 177 | 186 | 177 | 180 | 160 | 166 | 134 | 150 | 169 | 169 |  | 1 | |
| niche4 | 150 | 153 | 176 | 179 | 186 | 189 | 177 | 180 | 160 | 170 | 142 | 150 | 169 | 173 |  | 1 | |
| niche5 | 150 | 153 | 176 | 179 | 186 | 189 | 174 | 180 | 160 | 160 | 134 | 150 | 165 | 169 |  | 1 | |
| niche6 | 153 | 165 | 170 | 179 | 186 | 189 | 174 | 180 | 160 | 170 | 150 | 150 | 165 | 169 |  | 1 | |
| niche7 | 153 | 165 | 167 | 179 | 189 | 189 | 171 | 177 | 160 | 170 | 142 | 150 | 165 | 169 |  | 1 | |
| niche8 | 153 | 153 | 167 | 179 | 186 | 186 | 171 | 177 | 158 | 162 | 142 | 150 | 165 | 169 |  | 1 | |
| niche9 | 150 | 165 | 167 | 179 | 186 | 186 | 177 | 180 | 160 | 160 | 142 | 150 | 169 | 173 |  | 1 | |
| niche10 | 150 | 165 | 176 | 179 | 186 | 189 | 177 | 180 | 160 | 166 | 142 | 150 | 169 | 173 |  | 1 | |
| niche11 | 153 | 153 | 176 | 179 | 186 | 189 | 171 | 177 | 158 | 162 | 134 | 150 | 169 | 173 |  | 1 | |
| niche12 | 150 | 153 | 170 | 179 | 186 | 186 | 171 | 177 | 158 | 162 | 134 | 150 | 165 | 169 |  | 1 | |
| niche13 | 150 | 150 | 176 | 179 | 186 | 186 | 177 | 180 | 160 | 166 | 134 | 150 | 166 | 173 |  | 1 | |
| niche14 | 150 | 150 | 176 | 179 | 186 | 189 | 177 | 180 | 160 | 166 | 142 | 150 | 165 | 169 |  | 1 | |
| niche15 | 150 | 153 | 176 | 179 | 186 | 189 | 177 | 180 | 160 | 166 | 134 | 150 | 165 | 169 |  | 1 | |
| niche16 | 153 | 153 | 176 | 179 | 186 | 189 | 171 | 177 | 158 | 162 | 142 | 150 | 165 | 169 |  | 1 | |
| niche17 | 150 | 150 | 167 | 179 | 186 | 186 | 174 | 180 | 160 | 170 | 134 | 150 | 165 | 169 |  | 1 | |
| niche18 | 150 | 153 | 176 | 179 | 189 | 189 | 171 | 177 | 158 | 166 | 142 | 150 | 169 | 173 |  | 1 | |
| niche19 | 165 | 165 | 167 | 179 | 189 | 189 | 171 | 177 | 158 | 166 | 142 | 150 | 165 | 169 |  | 1 | |
| niche20 | 150 | 165 | 170 | 179 | 186 | 186 | 171 | 177 | 162 | 162 | 142 | 150 | 169 | 173 |  | 1 | |
| niche21 | 150 | 150 | 170 | 179 | 186 | 189 | 171 | 177 | 162 | 162 | 142 | 150 | 169 | 173 |  | 1 | |
| niche22 | 150 | 150 | 170 | 179 | 186 | 189 | 180 | 180 | 160 | 160 | 150 | 150 | 165 | 169 |  | 1 | |
| niche23 | 165 | 165 | 170 | 179 | 186 | 186 | 171 | 177 | 160 | 170 | 134 | 150 | 165 | 169 |  | 1 | |
| niche24 | 165 | 165 | 167 | 179 | 186 | 189 | 174 | 180 | 162 | 162 | 142 | 150 | 165 | 169 |  | 1 | |
| niche25 | 150 | 165 | 176 | 179 | 186 | 189 | 174 | 180 | 160 | 170 | 142 | 150 | 169 | 173 |  | 1 | |
| niche26 | 153 | 153 | 176 | 179 | 180 | 183 | 174 | 180 | 160 | 170 | 142 | 150 | 169 | 169 |  | 1 | |
| niche27 | 150 | 150 | 176 | 179 | 180 | 180 | 171 | 177 | 162 | 162 | 142 | 150 | 169 | 169 |  | 1 | |
| niche28 | 153 | 165 | 176 | 179 | 180 | 180 | 171 | 177 | 162 | 162 | 142 | 150 | 165 | 169 |  | 1 | |
| niche29 | 150 | 150 | 170 | 179 | 180 | 183 | 174 | 180 | 160 | 170 | 142 | 150 | 169 | 169 |  | 1 | |
| niche30 | 153 | 165 | 170 | 179 | 180 | 180 | 177 | 180 | 162 | 166 | 142 | 150 | 169 | 169 |  | 1 | |
| niche31 | 165 | 165 | 167 | 179 | 177 | 186 | 171 | 180 | 160 | 166 | 142 | 150 | 169 | 173 |  | 1 | |
| niche32 | 150 | 153 | 176 | 179 | 177 | 186 | 171 | 177 | 160 | 166 | 134 | 150 | 169 | 173 |  | 1 | |
| niche33 | 153 | 153 | 176 | 179 | 186 | 189 | 174 | 180 | 156 | 160 | 142 | 150 | 169 | 173 |  | 1 | |
| niche34 | 165 | 165 | 167 | 179 | 186 | 189 | 174 | 180 | 156 | 160 | 142 | 150 | 169 | 173 |  | 1 | |
| niche35 | 165 | 165 | 167 | 179 | 186 | 186 | 177 | 180 | 156 | 160 | 142 | 150 | 169 | 173 |  | 1 | |
| niche36 | 153 | 153 | 167 | 179 | 189 | 189 | 177 | 180 | 160 | 166 | 142 | 150 | 169 | 173 |  | 1 | |
| niche37 | 153 | 153 | 167 | 179 | 189 | 189 | 177 | 180 | 160 | 166 | 134 | 150 | 169 | 169 |  | 1 | |
| niche38 | 150 | 153 | 167 | 179 | 186 | 189 | 174 | 180 | 156 | 160 | 142 | 150 | 169 | 173 |  | 1 | |
| niche39 | 165 | 165 | 176 | 179 | 186 | 189 | 174 | 180 | 160 | 166 | 134 | 150 | 165 | 173 |  | 1 | |
| niche40 | 165 | 165 | 167 | 179 | 189 | 189 | 174 | 180 | 160 | 166 | 134 | 150 | 169 | 173 |  | 1 | |
| niche41 | 150 | 150 | 176 | 179 | 186 | 189 | 177 | 180 | 156 | 166 | 142 | 150 | 169 | 173 |  | 1 | |
| niche42 | 150 | 153 | 176 | 179 | 186 | 189 | 177 | 180 | 156 | 166 | 142 | 150 | 169 | 173 |  | 1 | |
| niche43 | 150 | 150 | 167 | 179 | 189 | 189 | 177 | 180 | 160 | 166 | 134 | 150 | 169 | 173 |  | 1 | |
| niche44 | 150 | 153 | 176 | 179 | 189 | 189 | 174 | 180 | 156 | 160 | 141 | 150 | 169 | 173 |  | 1 | |
| niche45 | 150 | 153 | 176 | 179 | 186 | 189 | 177 | 180 | 156 | 160 | 141 | 150 | 169 | 173 |  | 1 | |
| niche47 | 150 | 150 | 176 | 179 | 189 | 189 | 177 | 180 | 156 | 160 | 141 | 150 | 169 | 173 |  | 1 | |
| niche48 | 150 | 153 | 176 | 179 | 189 | 189 | 177 | 180 | 156 | 160 | 141 | 150 | 169 | 173 |  | 2 | |
| niche49 | 153 | 153 | 176 | 179 | 189 | 189 | 177 | 180 | 156 | 160 | 141 | 150 | 169 | 173 |  | 1 | |
| niche50 | 153 | 153 | 176 | 179 | 189 | 189 | 177 | 180 | 156 | 166 | 142 | 146 | 165 | 169 |  | 1 | |
| niche51 | 153 | 153 | 176 | 179 | 189 | 189 | 177 | 180 | 162 | 166 | 134 | 150 | 165 | 171 |  | 1 | |
| talqu1 | 150 | 165 | 176 | 179 | 180 | 183 | 177 | 180 | 162 | 166 | 150 | 164 | 169 | 169 |  | 1 | |
| talqu2 | 153 | 165 | 176 | 179 | 180 | 189 | 174 | 180 | 160 | 160 | 150 | 164 | 171 | 171 |  | 1 | |
| talqu3 | 150 | 153 | 176 | 179 | 177 | 186 | 180 | 180 | 160 | 160 | 142 | 150 | 171 | 171 |  | 1 | |
| talqu4 | 153 | 165 | 167 | 179 | 180 | 180 | 177 | 180 | 164 | 166 | 142 | 150 | 169 | 173 |  | 1 | |
| talqu5 | 153 | 165 | 176 | 179 | 180 | 189 | 180 | 180 | 164 | 166 | 150 | 164 | 169 | 173 |  | 1 | |
| talqu6 | 150 | 150 | 170 | 179 | 180 | 189 | 174 | 180 | 164 | 164 | 150 | 164 | 169 | 173 |  | 1 | |
| talqu7 | 150 | 150 | 167 | 179 | 180 | 189 | 174 | 174 | 160 | 170 | 142 | 150 | 171 | 173 |  | 1 | |
| talqu8 | 150 | 150 | 176 | 179 | 174 | 189 | 160 | 166 | 160 | 166 | 142 | 150 | 165 | 171 |  | 1 | |
| talqu9 | 150 | 150 | 167 | 179 | 174 | 189 | 174 | 180 | 160 | 170 | 150 | 150 | 169 | 169 |  | 1 | |
| talqu10 | 150 | 153 | 170 | 179 | 180 | 183 | 171 | 177 | 156 | 162 | 142 | 150 | 169 | 173 |  | 1 | |
| talqu11 | 150 | 150 | 176 | 179 | 180 | 189 | 174 | 180 | 160 | 170 | 150 | 164 | 171 | 173 |  | 1 | |
| talqu12 | 165 | 165 | 176 | 179 | 180 | 183 | 174 | 180 | 166 | 166 | 142 | 150 | 171 | 173 |  | 1 | |
| talqu13 | 150 | 150 | 170 | 179 | 180 | 189 | 171 | 177 | 160 | 166 | 150 | 150 | 171 | 173 |  | 1 | |
| talqu14 | 150 | 150 | 176 | 179 | 180 | 189 | 174 | 180 | 160 | 160 | 142 | 150 | 171 | 173 |  | 1 | |
| talqu15 | 150 | 150 | 170 | 179 | 180 | 183 | 174 | 180 | 166 | 166 | 150 | 150 | 169 | 171 |  | 1 | |
| talqu16 | 150 | 153 | 176 | 179 | 180 | 189 | 174 | 180 | 160 | 170 | 150 | 150 | 171 | 171 |  | 1 | |
| talqu17 | 150 | 153 | 167 | 179 | 186 | 189 | 174 | 180 | 160 | 166 | 150 | 150 | 171 | 171 |  | 1 | |
| talqu18 | 150 | 150 | 167 | 179 | 180 | 189 | 174 | 180 | 166 | 170 | 150 | 150 | 169 | 173 |  | 1 | |
| talqu19 | 150 | 150 | 176 | 179 | 180 | 189 | 171 | 177 | 166 | 166 | 142 | 164 | 169 | 173 |  | 1 | |
| talqu20 | 150 | 150 | 176 | 179 | 180 | 189 | 171 | 177 | 162 | 166 | 142 | 164 | 165 | 165 |  | 1 | |
| talqu22 | 150 | 150 | 176 | 179 | 180 | 189 | 171 | 171 | 166 | 166 | 150 | 164 | 165 | 173 |  | 1 | |
| talqu23 | 150 | 165 | 176 | 179 | 180 | 189 | 171 | 177 | 166 | 166 | 150 | 164 | 165 | 165 |  | 1 | |
| talqu24 | 153 | 153 | 176 | 179 | 180 | 180 | 174 | 180 | 166 | 166 | 150 | 164 | 165 | 165 |  | 1 | |
| talqu25 | 153 | 153 | 176 | 179 | 180 | 180 | 171 | 171 | 162 | 166 | 142 | 150 | 165 | 173 |  | 1 | |
| talqu26 | 150 | 150 | 176 | 179 | 180 | 180 | 171 | 171 | 166 | 166 | 150 | 165 | 165 | 173 |  | 1 | |
| talqu27 | 150 | 150 | 176 | 179 | 180 | 189 | 171 | 171 | 162 | 166 | 150 | 164 | 165 | 173 |  | 1 | |
| talqu28 | 150 | 150 | 176 | 179 | 180 | 180 | 174 | 180 | 162 | 166 | 150 | 164 | 165 | 173 |  | 1 | |
| talqu29 | 150 | 150 | 176 | 179 | 180 | 180 | 171 | 177 | 162 | 166 | 150 | 164 | 165 | 165 |  | 2 | |
| talqu30 | 150 | 150 | 176 | 179 | 174 | 174 | 171 | 171 | 166 | 166 | 150 | 164 | 165 | 173 |  | 1 | |
| talqu31 | 150 | 150 | 176 | 179 | 180 | 180 | 171 | 171 | 162 | 166 | 150 | 164 | 165 | 173 |  | 1 | |
| talqu32 | 150 | 150 | 167 | 179 | 180 | 189 | 174 | 180 | 162 | 166 | 150 | 164 | 169 | 173 |  | 1 | |
| talqu33 | 150 | 150 | 176 | 179 | 180 | 180 | 171 | 171 | 162 | 166 | 142 | 164 | 165 | 173 |  | 1 | |
| talqu34 | 150 | 150 | 176 | 179 | 180 | 180 | 171 | 177 | 166 | 170 | 150 | 164 | 165 | 165 |  | 1 | |
| talqu35 | 150 | 150 | 176 | 179 | 180 | 180 | 171 | 171 | 166 | 170 | 150 | 164 | 165 | 173 |  | 1 | |
| talqu36 | 150 | 150 | 176 | 179 | 174 | 174 | 174 | 180 | 166 | 170 | 150 | 164 | 165 | 173 |  | 1 | |
| mirar1 | 150 | 150 | 167 | 170 | 174 | 174 | 174 | 180 | 166 | 170 | 142 | 164 | 169 | 173 |  | 1 | |
| mirar2 | 150 | 150 | 167 | 170 | 174 | 174 | 174 | 180 | 160 | 170 | 142 | 164 | 169 | 173 |  | 1 | |
| mirar3 | 153 | 153 | 167 | 170 | 174 | 174 | 174 | 180 | 162 | 166 | 142 | 164 | 169 | 173 |  | 1 | |
| mirar4 | 153 | 153 | 167 | 170 | 177 | 186 | 174 | 180 | 162 | 166 | 142 | 164 | 169 | 173 |  | 1 | |
| mirar5 | 150 | 150 | 167 | 170 | 180 | 189 | 174 | 180 | 162 | 170 | 142 | 164 | 165 | 169 |  | 1 | |
| mirar6 | 150 | 150 | 176 | 179 | 180 | 180 | 174 | 180 | 160 | 170 | 142 | 150 | 165 | 173 |  | 1 | |
| mirar7 | 153 | 153 | 176 | 179 | 186 | 195 | 174 | 180 | 160 | 170 | 150 | 150 | 165 | 165 |  | 1 | |
| mirar8 | 153 | 153 | 176 | 179 | 186 | 195 | 174 | 180 | 160 | 170 | 140 | 150 | 165 | 173 |  | 1 | |
| mirar9 | 150 | 150 | 176 | 179 | 186 | 186 | 171 | 177 | 160 | 170 | 144 | 150 | 165 | 165 |  | 1 | |
| mirar10 | 153 | 153 | 176 | 179 | 189 | 189 | 165 | 171 | 162 | 166 | 150 | 164 | 165 | 173 |  | 1 | |
| mirar11 | 153 | 153 | 176 | 179 | 186 | 186 | 165 | 171 | 162 | 166 | 150 | 164 | 165 | 165 |  | 1 | |
| mirar12 | 153 | 153 | 176 | 179 | 186 | 186 | 171 | 177 | 160 | 170 | 150 | 164 | 165 | 169 |  | 1 | |
| mirar13 | 153 | 153 | 176 | 179 | 186 | 195 | 171 | 177 | 160 | 170 | 150 | 164 | 165 | 169 |  | 1 | |
| mirar14 | 150 | 150 | 167 | 179 | 186 | 195 | 174 | 180 | 160 | 170 | 150 | 164 | 165 | 169 |  | 1 | |
| mirar15 | 153 | 153 | 176 | 179 | 186 | 195 | 165 | 171 | 162 | 166 | 150 | 164 | 165 | 173 |  | 1 | |
| mirar16 | 153 | 153 | 167 | 179 | 186 | 189 | 174 | 180 | 160 | 170 | 150 | 164 | 165 | 173 |  | 1 | |
| mirar17 | 153 | 165 | 167 | 179 | 186 | 195 | 174 | 180 | 162 | 166 | 150 | 164 | 160 | 165 |  | 1 | |
| mirar18 | 153 | 153 | 176 | 179 | 186 | 186 | 165 | 171 | 162 | 166 | 150 | 164 | 160 | 165 |  | 1 | |
| mirar19 | 153 | 165 | 167 | 179 | 186 | 195 | 171 | 177 | 162 | 166 | 150 | 164 | 160 | 165 |  | 1 | |
| mirar20 | 153 | 153 | 167 | 179 | 186 | 198 | 174 | 180 | 162 | 166 | 150 | 164 | 165 | 165 |  | 1 | |
| mirar21 | 153 | 153 | 167 | 179 | 186 | 195 | 171 | 177 | 162 | 166 | 150 | 164 | 165 | 165 |  | 1 | |
| mirar24 | 165 | 165 | 167 | 179 | 186 | 195 | 171 | 177 | 162 | 166 | 150 | 164 | 165 | 169 |  | 1 | |
| mirar25 | 165 | 165 | 167 | 179 | 186 | 195 | 171 | 177 | 162 | 166 | 150 | 164 | 165 | 165 |  | 1 | |
| mirar26 | 165 | 165 | 167 | 179 | 186 | 195 | 171 | 177 | 156 | 160 | 150 | 164 | 165 | 165 |  | 1 | |
| mirar27 | 153 | 153 | 167 | 179 | 186 | 195 | 171 | 177 | 156 | 166 | 150 | 164 | 165 | 169 |  | 1 | |
| mirar28 | 153 | 153 | 176 | 179 | 186 | 186 | 171 | 177 | 160 | 166 | 128 | 142 | 169 | 173 |  | 1 | |
| cato6 | 153 | 165 | 176 | 179 | 186 | 195 | 174 | 180 | 160 | 170 | 142 | 146 | 169 | 173 |  | 1 | |
| cato15 | 150 | 156 | 176 | 179 | 180 | 189 | 171 | 180 | 160 | 170 | 142 | 150 | 165 | 169 |  | 1 | |
| cato17 | 153 | 153 | 176 | 179 | 180 | 189 | 174 | 180 | 160 | 170 | 142 | 150 | 165 | 169 |  | 1 | |
| cato19 | 153 | 153 | 176 | 179 | 180 | 189 | 171 | 180 | 160 | 170 | 142 | 150 | 165 | 169 |  | 13 | |
| cato21 | 153 | 153 | 176 | 179 | 180 | 189 | 171 | 180 | 160 | 166 | 142 | 150 | 169 | 169 |  | 3 | |
| cato23 | 153 | 153 | 176 | 179 | 180 | 189 | 171 | 180 | 160 | 166 | 142 | 150 | 165 | 169 |  | 4 | |
| cato24 | 153 | 153 | 176 | 179 | 180 | 189 | 171 | 180 | 160 | 160 | 142 | 150 | 165 | 169 |  | 1 | |
| cato26 | 153 | 153 | 176 | 179 | 186 | 189 | 171 | 180 | 160 | 160 | 142 | 150 | 165 | 169 |  | 1 | |
| cato27 | 153 | 153 | 176 | 179 | 186 | 189 | 171 | 180 | 166 | 166 | 142 | 150 | 165 | 169 |  | 1 | |
| cato28 | 153 | 153 | 167 | 179 | 186 | 189 | 171 | 180 | 162 | 162 | 142 | 150 | 165 | 169 |  | 1 | |
| cato29 | 150 | 150 | 167 | 179 | 186 | 189 | 177 | 180 | 162 | 162 | 142 | 150 | 173 | 173 |  | 1 | |
| cato30 | 153 | 165 | 167 | 179 | 186 | 189 | 177 | 180 | 160 | 166 | 142 | 150 | 165 | 169 |  | 1 | |
| mulch1 | 165 | 165 | 167 | 179 | 186 | 189 | 177 | 180 | 162 | 166 | 142 | 150 | 165 | 169 |  | 1 | |
| mulch2 | 150 | 150 | 167 | 179 | 180 | 189 | 177 | 180 | 162 | 166 | 142 | 150 | 169 | 169 |  | 1 | |
| mulch3 | 150 | 165 | 167 | 179 | 177 | 186 | 177 | 180 | 162 | 166 | 142 | 150 | 169 | 169 |  | 1 | |
| mulch4 | 150 | 165 | 167 | 179 | 177 | 189 | 177 | 180 | 160 | 160 | 142 | 150 | 169 | 173 |  | 1 | |
| mulch5 | 150 | 150 | 167 | 179 | 186 | 189 | 177 | 180 | 160 | 166 | 142 | 150 | 165 | 169 |  | 1 | |
| mulch6 | 153 | 165 | 167 | 179 | 186 | 189 | 177 | 180 | 160 | 160 | 142 | 150 | 171 | 171 |  | 1 | |
| mulch7 | 153 | 165 | 167 | 179 | 186 | 189 | 177 | 180 | 160 | 166 | 142 | 150 | 173 | 173 |  | 1 | |
| mulch8 | 153 | 153 | 176 | 179 | 177 | 189 | 177 | 180 | 160 | 166 | 142 | 150 | 173 | 173 |  | 1 | |
| mulch9 | 150 | 150 | 176 | 179 | 177 | 177 | 177 | 180 | 160 | 162 | 142 | 150 | 173 | 173 |  | 1 | |
| mulch10 | 150 | 165 | 167 | 179 | 177 | 189 | 174 | 180 | 160 | 162 | 150 | 164 | 165 | 172 |  | 1 | |
| mulch11 | 150 | 165 | 179 | 179 | 186 | 189 | 174 | 180 | 160 | 166 | 134 | 150 | 173 | 173 |  | 1 | |
| mulch12 | 153 | 165 | 179 | 179 | 186 | 186 | 174 | 180 | 150 | 164 | 150 | 164 | 169 | 173 |  | 1 | |
| mulch13 | 150 | 165 | 179 | 179 | 186 | 189 | 177 | 180 | 166 | 166 | 134 | 150 | 173 | 173 |  | 1 | |
| mulch16 | 150 | 150 | 179 | 179 | 186 | 189 | 174 | 180 | 160 | 162 | 142 | 150 | 173 | 173 |  | 1 | |
| mulch17 | 150 | 150 | 179 | 179 | 186 | 189 | 177 | 180 | 166 | 166 | 142 | 150 | 165 | 169 |  | 1 | |
| mulch18 | 150 | 150 | 179 | 179 | 186 | 189 | 177 | 180 | 160 | 162 | 150 | 164 | 165 | 173 |  | 1 | |
| mulch19 | 150 | 150 | 179 | 179 | 186 | 189 | 177 | 180 | 166 | 166 | 150 | 164 | 169 | 173 |  | 1 | |
| mulch20 | 150 | 150 | 179 | 179 | 186 | 189 | 177 | 180 | 160 | 162 | 142 | 150 | 173 | 173 |  | 3 | |
| mulch21 | 150 | 150 | 179 | 179 | 186 | 189 | 177 | 180 | 160 | 166 | 142 | 150 | 173 | 173 |  | 1 | |
| mulch22 | 150 | 150 | 167 | 170 | 186 | 189 | 177 | 180 | 160 | 166 | 142 | 150 | 173 | 173 |  | 1 | |
| mulch23 | 150 | 156 | 167 | 170 | 186 | 189 | 177 | 180 | 160 | 166 | 142 | 150 | 165 | 169 |  | 1 | |
| mulch24 | 153 | 165 | 176 | 179 | 186 | 189 | 174 | 180 | 160 | 166 | 142 | 150 | 165 | 169 |  | 1 | |
| mulch25 | 150 | 165 | 176 | 179 | 186 | 189 | 177 | 180 | 160 | 166 | 134 | 150 | 173 | 173 |  | 1 | |
| mulch26 | 150 | 165 | 176 | 179 | 186 | 186 | 177 | 180 | 160 | 166 | 142 | 150 | 173 | 173 |  | 1 | |
| mulch27 | 150 | 156 | 176 | 179 | 186 | 189 | 177 | 180 | 160 | 162 | 142 | 150 | 165 | 169 |  | 1 | |
| mulch28 | 150 | 156 | 176 | 179 | 186 | 189 | 177 | 180 | 160 | 166 | 132 | 150 | 173 | 173 |  | 1 | |
| patag1 | 150 | 153 | 176 | 179 | 186 | 189 | 174 | 180 | 160 | 166 | 132 | 142 | 171 | 173 |  | 1 | |
| patag5 | 150 | 153 | 176 | 179 | 186 | 189 | 171 | 180 | 160 | 166 | 142 | 150 | 171 | 173 |  | 4 | |
| patag9 | 150 | 150 | 176 | 179 | 186 | 189 | 177 | 180 | 160 | 166 | 132 | 142 | 171 | 173 |  | 3 | |
| patag10 | 150 | 150 | 176 | 179 | 186 | 189 | 171 | 180 | 160 | 166 | 132 | 142 | 165 | 169 |  | 1 | |
| patag13 | 150 | 150 | 176 | 179 | 186 | 189 | 171 | 180 | 166 | 166 | 132 | 142 | 171 | 173 |  | 1 | |
| patag14 | 150 | 150 | 176 | 179 | 186 | 189 | 171 | 180 | 160 | 166 | 132 | 142 | 171 | 173 |  | 4 | |
| patag15 | 150 | 150 | 167 | 179 | 186 | 189 | 180 | 180 | 160 | 166 | 132 | 142 | 165 | 173 |  | 1 | |
| patag16 | 150 | 150 | 176 | 179 | 186 | 189 | 177 | 177 | 160 | 166 | 132 | 142 | 171 | 173 |  | 1 | |
| patag18 | 153 | 153 | 176 | 179 | 177 | 186 | 174 | 180 | 156 | 166 | 142 | 150 | 171 | 173 |  | 1 | |
| patag20 | 150 | 150 | 176 | 179 | 186 | 189 | 174 | 180 | 160 | 166 | 142 | 150 | 165 | 169 |  | 2 | |
| patag23 | 150 | 150 | 176 | 179 | 186 | 189 | 174 | 180 | 160 | 166 | 142 | 150 | 171 | 173 |  | 4 | |
| patag24 | 150 | 150 | 167 | 179 | 186 | 189 | 174 | 180 | 160 | 166 | 132 | 142 | 171 | 173 |  | 2 | |
| patag25 | 150 | 150 | 176 | 179 | 186 | 189 | 174 | 180 | 160 | 166 | 132 | 142 | 165 | 173 |  | 1 | |
| patag26 | 150 | 150 | 176 | 179 | 177 | 186 | 174 | 180 | 160 | 166 | 132 | 142 | 165 | 173 |  | 1 | |
| patag27 | 150 | 150 | 167 | 179 | 177 | 186 | 174 | 180 | 156 | 160 | 142 | 150 | 171 | 173 |  | 1 | |
| patag28 | 153 | 153 | 167 | 179 | 186 | 189 | 174 | 180 | 156 | 160 | 142 | 150 | 171 | 173 |  | 1 | |
| patag29 | 153 | 153 | 176 | 179 | 186 | 189 | 174 | 180 | 160 | 166 | 142 | 150 | 169 | 173 |  | 1 | |
| patag31 | 150 | 150 | 176 | 179 | 177 | 186 | 174 | 180 | 160 | 166 | 142 | 150 | 169 | 173 |  | 1 | |
| canad1 | 150 | 150 | 176 | 179 | 186 | 189 | 174 | 180 | 160 | 166 | 142 | 150 | 165 | 173 |  | 1 | |
| canad2 | 150 | 150 | 176 | 179 | 177 | 186 | 174 | 180 | 160 | 166 | 150 | 164 | 169 | 173 |  | 1 | |
| canad3 | 153 | 153 | 176 | 179 | 177 | 189 | 174 | 180 | 160 | 166 | 142 | 150 | 169 | 173 |  | 1 | |
| canad4 | 150 | 150 | 176 | 179 | 177 | 189 | 174 | 177 | 156 | 166 | 134 | 150 | 169 | 173 |  | 1 | |
| canad5 | 153 | 153 | 176 | 179 | 177 | 189 | 174 | 177 | 160 | 166 | 134 | 150 | 169 | 173 |  | 1 | |
| canad6 | 150 | 150 | 176 | 179 | 186 | 189 | 174 | 177 | 156 | 166 | 142 | 150 | 171 | 173 |  | 1 | |
| canad7 | 150 | 153 | 176 | 179 | 177 | 189 | 174 | 180 | 160 | 166 | 142 | 150 | 168 | 173 |  | 1 | |
| canad8 | 150 | 150 | 176 | 179 | 177 | 189 | 174 | 177 | 156 | 166 | 142 | 150 | 168 | 173 |  | 1 | |
| canad9 | 150 | 153 | 176 | 179 | 177 | 189 | 174 | 180 | 166 | 166 | 142 | 150 | 168 | 173 |  | 1 | |
| canad10 | 150 | 165 | 176 | 179 | 177 | 189 | 174 | 177 | 156 | 166 | 142 | 150 | 165 | 169 |  | 1 | |
| canad11 | 150 | 150 | 176 | 179 | 177 | 189 | 174 | 177 | 156 | 166 | 142 | 150 | 169 | 173 |  | 1 | |
| canad12 | 150 | 150 | 176 | 179 | 177 | 189 | 174 | 180 | 160 | 166 | 142 | 150 | 165 | 169 |  | 1 | |
| canad13 | 150 | 165 | 176 | 179 | 177 | 189 | 174 | 177 | 160 | 166 | 134 | 150 | 169 | 173 |  | 1 | |
| pang1 | 153 | 165 | 176 | 179 | 177 | 189 | 180 | 180 | 160 | 166 | 142 | 150 | 169 | 171 |  | 1 | |
| pang2 | 150 | 153 | 176 | 179 | 186 | 189 | 180 | 180 | 160 | 166 | 134 | 150 | 171 | 173 |  | 1 | |
| pang3 | 153 | 165 | 176 | 179 | 189 | 189 | 180 | 180 | 160 | 166 | 144 | 150 | 171 | 173 |  | 1 | |
| pang4 | 153 | 165 | 176 | 179 | 186 | 189 | 180 | 180 | 160 | 166 | 142 | 150 | 171 | 173 |  | 1 | |
| pang5 | 150 | 150 | 176 | 179 | 177 | 189 | 180 | 180 | 160 | 166 | 134 | 150 | 171 | 173 |  | 1 | |
| pang6 | 150 | 165 | 176 | 179 | 189 | 189 | 171 | 174 | 160 | 166 | 134 | 150 | 169 | 173 |  | 1 | |
| pang7 | 150 | 165 | 176 | 179 | 189 | 189 | 180 | 180 | 164 | 170 | 134 | 150 | 169 | 171 |  | 1 | |
| pang8 | 150 | 165 | 176 | 179 | 186 | 189 | 180 | 180 | 160 | 166 | 142 | 150 | 169 | 171 |  | 1 | |
| pang9 | 150 | 165 | 176 | 179 | 189 | 189 | 174 | 180 | 160 | 166 | 142 | 150 | 169 | 171 |  | 1 | |
| pang11 | 153 | 153 | 176 | 179 | 189 | 189 | 174 | 180 | 160 | 166 | 142 | 150 | 165 | 169 |  | 2 | |
| pang12 | 153 | 165 | 176 | 179 | 186 | 189 | 171 | 180 | 166 | 166 | 142 | 150 | 165 | 169 |  | 1 | |
| pang13 | 150 | 165 | 176 | 179 | 189 | 189 | 174 | 180 | 162 | 166 | 142 | 150 | 169 | 171 |  | 1 | |
| pang16 | 153 | 165 | 176 | 179 | 186 | 189 | 174 | 180 | 160 | 166 | 150 | 164 | 169 | 171 |  | 1 | |
| pang17 | 165 | 165 | 176 | 179 | 177 | 186 | 174 | 180 | 160 | 166 | 148 | 164 | 165 | 169 |  | 1 | |
| pang19 | 150 | 165 | 176 | 179 | 177 | 189 | 174 | 180 | 160 | 166 | 142 | 150 | 169 | 171 |  | 5 | |
| pang20 | 150 | 165 | 170 | 176 | 186 | 189 | 174 | 180 | 160 | 166 | 148 | 150 | 169 | 171 |  | 1 | |
| pang21 | 150 | 165 | 176 | 179 | 177 | 189 | 174 | 180 | 162 | 166 | 142 | 150 | 169 | 171 |  | 1 | |
| pang22 | 153 | 165 | 176 | 179 | 186 | 186 | 174 | 180 | 160 | 166 | 142 | 150 | 165 | 169 |  | 1 | |
| pang23 | 153 | 153 | 176 | 179 | 189 | 189 | 174 | 180 | 162 | 166 | 142 | 150 | 169 | 173 |  | 1 | |
| pang24 | 150 | 150 | 176 | 179 | 189 | 195 | 174 | 180 | 160 | 166 | 142 | 150 | 169 | 171 |  | 1 | |
| pang25 | 153 | 165 | 176 | 179 | 186 | 189 | 174 | 180 | 162 | 166 | 134 | 150 | 169 | 171 |  | 1 | |
| pang26 | 153 | 165 | 176 | 179 | 177 | 189 | 174 | 180 | 160 | 166 | 134 | 150 | 165 | 169 |  | 1 | |
| pang27 | 153 | 165 | 176 | 179 | 174 | 186 | 174 | 180 | 162 | 166 | 134 | 150 | 165 | 171 |  | 1 | |
| pang28 | 165 | 165 | 176 | 179 | 177 | 189 | 174 | 180 | 160 | 162 | 134 | 150 | 165 | 169 |  | 1 | |
| ssidr1 | 153 | 165 | 176 | 179 | 186 | 189 | 174 | 180 | 156 | 166 | 134 | 150 | 171 | 173 |  | 1 | |
| ssidr2 | 153 | 153 | 176 | 179 | 186 | 189 | 177 | 180 | 156 | 166 | 134 | 150 | 171 | 173 |  | 1 | |
| ssidr6 | 153 | 153 | 176 | 179 | 186 | 189 | 177 | 180 | 156 | 166 | 142 | 150 | 169 | 173 |  | 1 | |
| ssidr8 | 153 | 153 | 176 | 179 | 186 | 189 | 177 | 180 | 156 | 166 | 134 | 150 | 165 | 173 |  | 1 | |
| ssidr9 | 153 | 153 | 176 | 179 | 186 | 189 | 177 | 180 | 156 | 166 | 134 | 150 | 169 | 173 |  | 5 | |
| ssidr10 | 153 | 153 | 176 | 179 | 186 | 189 | 177 | 180 | 160 | 166 | 134 | 150 | 169 | 173 |  | 1 | |
| ssidr13 | 153 | 153 | 176 | 179 | 186 | 189 | 177 | 180 | 156 | 160 | 134 | 150 | 165 | 169 |  | 1 | |
| ssidr14 | 150 | 153 | 176 | 179 | 186 | 189 | 177 | 180 | 156 | 160 | 134 | 150 | 173 | 269 |  | 1 | |
| ssidr15 | 153 | 153 | 176 | 179 | 186 | 189 | 177 | 180 | 156 | 160 | 134 | 150 | 169 | 173 |  | 3 | |
| ssidr16 | 153 | 153 | 170 | 176 | 186 | 189 | 177 | 180 | 162 | 166 | 134 | 150 | 169 | 173 |  | 1 | |
| ssidr17 | 150 | 153 | 170 | 176 | 186 | 189 | 177 | 180 | 162 | 166 | 134 | 150 | 165 | 173 |  | 1 | |
| ssidr18 | 150 | 153 | 176 | 179 | 186 | 189 | 177 | 180 | 162 | 166 | 150 | 164 | 165 | 173 |  | 1 | |
| ssidr19 | 150 | 153 | 176 | 179 | 186 | 189 | 165 | 171 | 162 | 166 | 150 | 164 | 165 | 169 |  | 1 | |
| p1 | 153 | 165 | 176 | 179 | 189 | 189 | 171 | 174 | 162 | 166 | 148 | 164 | 165 | 169 |  | 1 | |
| p2 | 150 | 153 | 170 | 179 | 189 | 189 | 156 | 165 | 162 | 166 | 142 | 150 | 165 | 169 |  | 1 | |
| p3 | 150 | 153 | 173 | 179 | 186 | 186 | 177 | 180 | 162 | 166 | 150 | 164 | 165 | 169 |  | 1 | |
| p4 | 150 | 165 | 173 | 179 | 189 | 189 | 177 | 180 | 160 | 170 | 142 | 150 | 169 | 173 |  | 1 | |
| p5 | 150 | 153 | 173 | 179 | 189 | 189 | 177 | 180 | 162 | 166 | 142 | 150 | 169 | 173 |  | 1 | |
| p6 | 150 | 153 | 173 | 179 | 189 | 189 | 174 | 180 | 162 | 166 | 150 | 164 | 169 | 173 |  | 1 | |
| p7 | 150 | 153 | 173 | 179 | 189 | 189 | 177 | 180 | 162 | 166 | 142 | 150 | 165 | 173 |  | 1 | |
| p8 | 150 | 153 | 173 | 179 | 186 | 189 | 177 | 180 | 162 | 166 | 142 | 150 | 169 | 173 |  | 1 | |
| p9 | 153 | 165 | 173 | 179 | 186 | 189 | 177 | 180 | 162 | 166 | 142 | 150 | 165 | 173 |  | 1 | |
| p10 | 153 | 165 | 173 | 179 | 186 | 189 | 177 | 180 | 160 | 166 | 142 | 150 | 165 | 173 |  | 1 | |
| p11 | 153 | 165 | 170 | 179 | 186 | 189 | 177 | 180 | 160 | 166 | 150 | 164 | 165 | 173 |  | 1 | |
| p12 | 153 | 165 | 170 | 179 | 186 | 189 | 177 | 180 | 160 | 166 | 150 | 164 | 169 | 173 |  | 1 | |
| p13 | 165 | 165 | 170 | 179 | 189 | 189 | 177 | 180 | 162 | 166 | 142 | 150 | 169 | 173 |  | 1 | |
| p14 | 150 | 153 | 170 | 179 | 186 | 189 | 177 | 180 | 162 | 166 | 150 | 164 | 169 | 173 |  | 1 | |
| p15 | 153 | 165 | 170 | 179 | 189 | 189 | 174 | 180 | 160 | 166 | 142 | 150 | 169 | 173 |  | 1 | |
| p16 | 150 | 153 | 176 | 179 | 186 | 186 | 177 | 180 | 164 | 166 | 142 | 150 | 165 | 173 |  | 1 | |
| p17 | 153 | 165 | 170 | 179 | 186 | 189 | 177 | 180 | 160 | 170 | 150 | 164 | 165 | 173 |  | 1 | |
| p18 | 153 | 153 | 176 | 179 | 186 | 189 | 177 | 180 | 164 | 166 | 142 | 150 | 165 | 173 |  | 1 | |
| p_19_colin | 153 | 153 | 176 | 179 | 186 | 189 | 159 | 165 | 160 | 160 | 142 | 150 | 165 | 173 |  | 1 | |
| p20 | 150 | 153 | 170 | 179 | 186 | 189 | 174 | 180 | 162 | 166 | 128 | 142 | 165 | 173 |  | 1 | |
| p21 | 150 | 153 | 170 | 179 | 186 | 189 | 159 | 165 | 162 | 166 | 128 | 142 | 169 | 173 |  | 1 | |
| p23 | 153 | 153 | 170 | 179 | 186 | 189 | 159 | 165 | 162 | 166 | 128 | 142 | 165 | 173 |  | 1 | |
| p25 | 153 | 153 | 170 | 179 | 186 | 189 | 159 | 165 | 162 | 166 | 128 | 142 | 169 | 173 |  | 3 | |
| p26 | 150 | 153 | 176 | 179 | 186 | 189 | 174 | 180 | 158 | 166 | 134 | 150 | 169 | 173 |  | 1 | |
| p27_manzanos | 153 | 165 | 176 | 179 | 186 | 189 | 174 | 180 | 160 | 166 | 134 | 150 | 165 | 173 |  | 1 | |
| p28 | 150 | 153 | 170 | 179 | 186 | 195 | 171 | 180 | 160 | 166 | 150 | 164 | 169 | 173 |  | 1 | |
| p29 | 153 | 153 | 170 | 179 | 186 | 189 | 174 | 180 | 162 | 166 | 128 | 142 | 169 | 173 |  | 1 | |
| p30 | 150 | 153 | 176 | 179 | 180 | 186 | 174 | 180 | 162 | 166 | 130 | 142 | 169 | 173 |  | 1 | |
| p31 | 150 | 153 | 170 | 179 | 186 | 195 | 174 | 180 | 160 | 170 | 150 | 164 | 165 | 169 |  | 1 | |
| p32_lasrastras | 150 | 153 | 170 | 179 | 180 | 186 | 177 | 180 | 160 | 170 | 142 | 150 | 165 | 169 |  | 1 | |
| p39 | 150 | 153 | 176 | 179 | 186 | 189 | 177 | 180 | 160 | 166 | 148 | 150 | 173 | 173 |  | 3 | |
| p40 | 150 | 153 | 176 | 179 | 186 | 189 | 177 | 180 | 160 | 166 | 128 | 164 | 165 | 173 |  | 1 | |
| p42 | 150 | 153 | 176 | 179 | 186 | 189 | 177 | 180 | 160 | 166 | 148 | 150 | 169 | 173 |  | 1 | |
| p44 | 153 | 153 | 176 | 179 | 186 | 189 | 177 | 180 | 160 | 166 | 149 | 150 | 169 | 173 |  | 1 | |
| p45 | 150 | 153 | 176 | 179 | 189 | 189 | 177 | 180 | 160 | 166 | 150 | 150 | 165 | 169 |  | 1 | |
| p46 | 150 | 153 | 176 | 179 | 186 | 189 | 177 | 180 | 160 | 166 | 148 | 150 | 165 | 173 |  | 4 | |
| p47_sanfernando | 150 | 165 | 176 | 179 | 189 | 189 | 171 | 180 | 162 | 166 | 148 | 150 | 165 | 173 |  | 1 | |

Data Subset Without Repeated Matching Multilocus Genotypes
